# ANXUR Receptor-Like Kinases Coordinate Cell Wall Integrity with Growth at the Pollen Tube Tip Via NADPH Oxidases

**DOI:** 10.1371/journal.pbio.1001719

**Published:** 2013-11-26

**Authors:** Aurélien Boisson-Dernier, Dmytro S. Lituiev, Anna Nestorova, Christina Maria Franck, Sharme Thirugnanarajah, Ueli Grossniklaus

**Affiliations:** 1 Institute of Plant Biology, University of Zürich, Zürich, Switzerland; 2 Zürich-Basel Plant Science Center, ETH Zürich, Zürich, Switzerland; Cornell University, United States of America

## Abstract

It has become increasingly apparent that the extracellular matrix (ECM), which in plants corresponds to the cell wall, can influence intracellular activities in ways that go far beyond their supposedly passive mechanical support. In plants, growing cells use mechanisms sensing cell wall integrity to coordinate cell wall performance with the internal growth machinery to avoid growth cessation or loss of integrity. How this coordination precisely works is unknown. Previously, we reported that in the tip-growing pollen tube the ANXUR receptor-like kinases (RLKs) of the CrRLK1L subfamily are essential to sustain growth without loss of cell wall integrity in *Arabidopsis*. Here, we show that over-expression of the ANXUR RLKs inhibits growth by over-activating exocytosis and the over-accumulation of secreted cell wall material. Moreover, the characterization of mutations in two partially redundant pollen-expressed NADPH oxidases coupled with genetic interaction studies demonstrate that the ANXUR RLKs function upstream of these NADPH oxidases. Using the H_2_O_2_-sensitive HyPer and the Ca^2+^-sensitive YC3.60 sensors in NADPH oxidase-deficient mutants, we reveal that NADPH oxidases generate tip-localized, pulsating H_2_O_2_ production that functions, possibly through Ca^2+^ channel activation, to maintain a steady tip-focused Ca^2+^ gradient during growth. Our findings support a model where ECM-sensing receptors regulate reactive oxygen species production, Ca^2+^ homeostasis, and exocytosis to coordinate ECM-performance with the internal growth machinery.

## Abstract 2

Pollen tubes grow extremely rapidly to effect fertilization in plants. ANXUR receptor-like kinases facilitate this growth by linking the intracellular growth machinery of pollen tubes to the status of the extracellular matrix via H_2_O_2_ and Ca^2+^ signaling.

## Author Summary

Tip-growing cells, such as plant root hairs and pollen tubes or fungal hyphae, are characterized by a tip-focused Ca^2+^ gradient. These tip-growing cells tightly coordinate the loosening and pressure-driven deformation of their extracellular matrix (ECM)—the cell wall in plant cells—by locally adding new membrane and cell wall materials. In pollen tubes, which grow at amazing speeds to effect fertilization in plants, a class of kinases called the ANXUR receptor-like kinases (RLKs) sense perturbations in cell wall integrity, and their loss leads to pollen tube rupture. Here, we gain new insights into the mechanism of cell wall surveillance by these RLKs in the model plant *Arabidopsis*. We show that over-expressing ANXUR RLKs over-activates exocytosis, causing an over-accumulation of secreted cell wall material that eventually leads to growth arrest. Moreover, we find that the ANXUR RLKs function upstream of NADPH oxidases, which are membrane-anchored enzymes that produce reactive oxygen species (ROS). Using H_2_O_2_- and Ca^2+^-sensitive reporters, we show that NADPH oxidases generate tip-localized H_2_O_2_ production, which is required to maintain a steady, tip-focused Ca^2+^ gradient that is essential for pollen tube growth. We postulate that ECM-sensing receptors, such as the ANXUR RLKs, regulate ROS production, Ca^2+^ homeostasis, and exocytosis to coordinate the status of the ECM with the cell's internal growth machinery.

## Introduction

It is well established that growing animal cells control the biogenesis, deposition, and remodeling of their extracellular matrix (ECM). *In vivo* the ECM contributes to the bulk, shape, and strength of many tissues and, therefore, plays a central role in development [1]. However, it is often underappreciated that the ECM also controls intracellular activities far beyond providing mechanical stability. For example, the ECM is under continuous cellular surveillance in order to monitor the loss of adhesion to the surrounding matrix, which leads to apoptosis. Consequently, disruption of signaling between the ECM and the cell is associated with tumorigenicity [2]. Similarly, growing plant cells direct the deposition of the primary cell wall (CW): the plants rigid, carbohydrate-rich ECM that resists turgor pressure, yet is flexible enough to allow cell expansion. Growing plant cells tightly coordinate the loosening and pressure-driven deformation of the CW with the addition of new membrane and CW materials through exocytosis. Thus, the cell must be kept informed about any environmental changes modifying the CW properties in order to avoid growth arrest or rupture. To circumvent these catastrophic scenarios, it has become increasingly evident that plant cells have developed mechanisms to sense CW integrity, which relay information about CW performance to the internal growth machinery. The molecular nature of this relay mechanism, however, remains largely unknown [3].

Since the first reports on THESEUS1 (THE1 [4]) and FERONIA (FER [5]), these *Arabidopsis* receptor-like kinases (RLKs) of the *Catharanthus roseus* RLK1-like subfamily (CrRLK1L) have received increasing attention as putative sensors that coordinate cellular growth and CW integrity (reviewed in [6]–[8]). How this coordination precisely works and which molecular players of the growth machinery are involved remained elusive, although Rho GTPases of plants (ROPs) and the production of NADPH oxidase-dependent reactive oxygen species (ROS) have emerged as putative downstream components. The role of NADPH oxidases, the ROS-producing enzymes that, based on their homology to the catalytic glycoprotein subunit of the mammalian phagocyte oxidase (gp91^phox^), are also called “respiratory burst oxidase homologues (Rboh)”, has been firmly established in various fundamental processes. These include localized lignin deposition [9], stomatal closure [10], pathogen responses [11], and root hair growth [12]. NADPH oxidases are plasma membrane (PM)-bound enzymes with six trans-membrane domains, an N-terminal region that contains EF-hands, and a C-terminal oxidase domain responsible for oxidizing O_2_ to produce superoxide radicals in the apoplast (reviewed in [13],[14]). The latter can quickly be dismutated, enzymatically or otherwise, into H_2_O_2_ that can freely diffuse back from the apoplast into the cytosol.

Connections between members of the CrRLK1L and NADPH oxidase families have been proposed or established for THE1 and FER, respectively. For example, *THE1* has been reported to be a positive regulator of CW damage-induced ROS production in seedlings, possibly through RbohD [15], while *FER* is both a negative regulator of H_2_O_2_ production in unchallenged leaves [16] and of ROS in guard cells [17]. Furthermore, in root hairs that elongate by tip-growth, FER is a positive regulator of ROS production through the ROP2-RbohC pathway [18]. Similar to the *rbohC* loss-of-function mutant (also called *root hair defective2* [*rhd2*]), disruption of *FER* leads to an impairment of ROS production and defective root hairs that burst [12],[18]. Disruption of the redundant CrRLK1Ls *ANXUR1* (*ANX1*) and *ANX2*, the two closest homologues of *FER*, triggers the rupture of pollen tubes (PTs), the tip-growing male gametophytes of flowering plants, resulting in male sterility [19],[20]. Similar to *fer* root hairs, *anx1 anx2* double mutant pollen form bulges and burst, failing to maintain their integrity during growth. This indicates that the FER and ANX RLKs could be cell-surface receptors that control CW integrity in tip-growing cells. In PTs, genetic evidence for the involvement of NADPH oxidases is lacking, but several studies have revealed a role for ROS during PT growth that remains to be precisely characterized. For example, it has been shown that either the use of ROS scavengers or the NADPH oxidase inhibitor diphenylene iodonium (DPI), or the down-regulation of a NADPH oxidase, reduces PT growth in tobacco [21]. In addition, the application of DPI at higher concentrations has also been reported to induce PT rupture in lily [22].

Because of the difficulty to image the dynamics of ROS production with good spatial and temporal resolution, and because of its multi-faceted impacts on CW properties and the activation of intracellular signaling, it is unknown how NADPH oxidase-dependent ROS control polar growth [23],[24]. It was first reported that RbohC/RHD2 is required for calcium influx via the stimulation of Ca^2+^ channels and for the generation of a tip-focused gradient of cytosolic free calcium [Ca^2+^]_cyt_, which is essential for polar growth [12],[25]. Later, Monshausen and colleagues reported that, under certain conditions, *rbohC* root hairs still display a tip-focused Ca^2+^ gradient, showing that *RbohC* was not essential for its establishment [26]. Moreover, they showed that artificially increasing or decreasing apoplastic ROS leads to growth cessation and root hair bursting, respectively, consistent with a role for ROS in regulating CW properties [27]. Finally, oscillations in apoplastic ROS levels just behind the tip were reported during root hair growth and correlated with growth rate, leading the authors to propose a model in which ROS rigidify the CW behind the tip, such that growth would be restricted to the tip [26]. However, due to the irreversible nature of the ROS-sensitive oxidation of the dye they used, the observed oscillations are unlikely to reflect the true nature of ROS dynamics [28]. Nonetheless, both models—namely the growth-promoting effect at the tip related to intracellular Ca^2+^ signaling and the growth-inhibiting effect behind the tip by rigidifying the CW—are not mutually exclusive as they could recruit different forms of ROS at different times and in different locations.

In this study we show that over-expression of the ANX RLKs inhibits PT growth by the over-activation of exocytosis and the over-accumulation of secreted membrane and CW materials. Genetic interaction studies coupled with a phenotypic characterization of loss-of-function mutants of two partially redundant, pollen-expressed NADPH oxidases, *RbohH* and *RbohJ*, demonstrate that the ANX RLKs function upstream of these NADPH oxidases. Furthermore, analyses of the genetically encoded H_2_O_2_-sensitive HyPer and Ca^2+^-sensitive YC3.60 sensors in NADPH oxidase-deficient pollen revealed that NADPH oxidases generate tip-localized, pulsating ROS that are responsible—possibly through activation of Ca^2+^ channels—for maintaining a steady, tip-focused Ca^2+^ gradient.

## Results

### The Functional ANX1-YFP and ANX2-YFP Fusions Inhibit Pollen Tube Growth

**Figure 1:**
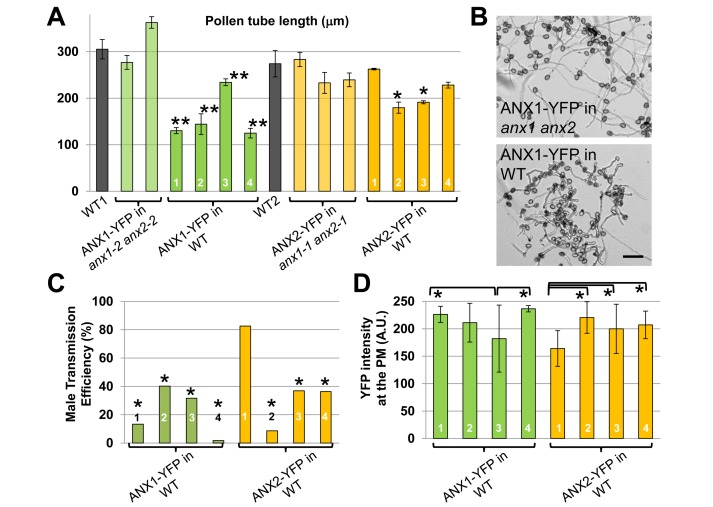
Over-expression of ANX RLKs inhibits pollen germination, pollen tube growth, and decreases male transmission efficiency. (A) Quantification of PT length after 5 h of *in vitro* growth for WT, two ANX1-YFP and three ANX2-YFP independent T3 complemented lines, and four independent T3 ANX1-YFP and ANX2-YFP over-expression lines. Data represent mean values ± standard error of the mean (SEM) of three independent experiments with more than 40 PTs per genotype and experiment. Single and double asterisks indicate significant differences from the WT according to a Student's *t* test with *p*<0.05 and *p*<0.01, respectively. See also Figure S1A. (B) Overview images of pollen of ANX1-YFP complemented and over-expression lines grown *in vitro* for 5 h. Scale bar = 100 µm. (C) TE_M_ of ANX1-YFP and ANX2-YFP for four independent over-expression lines. For each independent over-expression line, heterozygous T2 plants were crossed as pollen donor to the WT. More than 250 seeds resulting from each cross were grown on MS plates containing hygromycin and resistance was scored. TE_M_ was calculated as 100*(resistant/sensitive) in percent. Asterisks denote significant difference from the expected 1∶1 ratio for normal transmission with *p*<0.0001 (two-tailed exact Fisher's test). (D) Quantification of YFP fluorescence at the apical PM of growing PTs for each ANX1-YFP and ANX2-YFP over-expression line. Data represent mean values ± standard deviation (SD) (*n*>19 tubes for each line). Asterisks indicate significant difference among each ANX1-YFP or each ANX2-YFP over-expressing line (one-way ANOVA test, *p*<0.01).

We have previously shown that ANX1-yellow fluorescent protein (YFP) and ANX2-YFP protein fusions are polarly localized in the PM at the tip of growing PTs in independent T1 transgenic *Arabidopsis* lines [19]. Although in T1 lines, which contain a mixture of untransformed and transformed pollen grains, no obvious fertilization-related phenotypes could be detected, *in vitro* pollen germination and growth assays of homozygous lines that carry a single insertion of the constructs in the T3 generation revealed that ANX1-YFP and, to a lesser extent, ANX2-YFP inhibit pollen germination and PT growth compared to the wild type (WT) (Figures 1A, 1B, and S1). To investigate whether these phenotypes are due to over-expression or non-functionality of the fusion proteins, we transformed *anx1-2/anx1-2 anx2-2/ANX2* and *anx1-1/anx1-1 anx2-1/ANX2* plants with ANX1-YFP and ANX2-YFP fusions, respectively. In all T1 *anx1-2/anx1-2 anx2-2/ANX2* lines expressing ANX1-YFP and *anx1-1/anx1-1 anx2-1/ANX2* expressing ANX2-YFP PT rupture was reduced compared to the corresponding untransformed genotype (). Moreover, in all T3 homozygous lines with good ANX1/2-YFP expression in the *anx1 anx2* double mutant background, pollen germination, PT rupture, and PT length was indistinguishable from the WT (Figures 1A, 1B, S1, and S2). Thus, both ANX1-YFP and ANX2-YFP fusion proteins are functional, and the phenotypes observed in WT pollen expressing these fusion proteins are due to over-expression. Hereafter, independent homozygous lines expressing the ANX-YFP fusion proteins in the *anx1 anx2* background will be called either complemented lines or ANX-YFP in *anx1 anx2*, while homozygous lines expressing the same fusion proteins in a WT background will be referred to as ANX-OX or ANX-YFP in WT.

Pollen of ANX-OX lines germinated poorly and produced shorter and wider PTs than pollen of either WT or complemented lines (Figure 1A, 1B, and S1). To check whether these *in vitro* phenotypes impact the fitness of PTs *in vivo*, the male transmission efficiency (TE_M_) was assayed for each of the ANX-OX lines. Male transmission of ANX-YFP fusions was significantly decreased for all but one ANX2-OX line, showing that PTs over-expressing ANX-YFP fusion proteins are not as competitive as untransformed WT PTs (Figure 1C). Interestingly, the difference in the severity of these phenotypes between ANX1-OX lines or between ANX2-OX lines nicely correlated with the difference in the level of YFP fluorescence imaged at the PM of growing ANX-OX PTs (Figure 1D). The two strongest ANX1-OX lines (#1 and #4) and one ANX1-YFP complemented line were selected for further investigations.

### Over-expression of ANX1-YFP Triggers Cell Wall Accumulation and Plasma Membrane Invagination Via Increased Exocytosis

**Figure 2:**
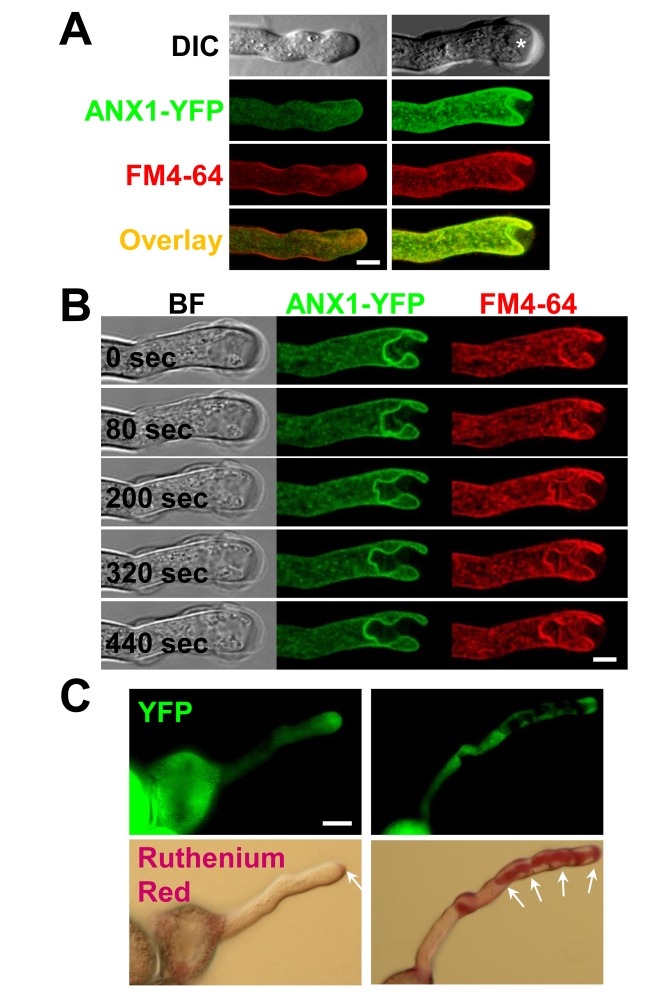
Over-expression of ANX RLKs triggers cell wall accumulation that leads to a cessation of pollen tube elongation and plasma membrane invagination. (A) Representative single median plane images of a normally growing PT of an ANX1-YFP complemented line (left) and an arrested PT of ANX1-YFP over-expressing line with apical membrane invagination (right). The different filters are indicated on the left. Before imaging, PTs were treated for 5 min with germination liquid medium containing FM4-64 (2 µM). Scale bar = 5 µm. (B) Time-course imaging of the apical PM invagination of an ANX1-YFP over-expressing PT that ceased to elongate. PTs were treated as in (A). See also corresponding Video S1. Filters are indicated at the top. Scale bar = 5 µm. (C) Representative bright-field and YFP fluorescence images of ANX1-YFP complemented (left) and over-expressing (right) PTs treated with 0.01% Ruthenium red, which stains acidic pectins. Note that staining is restricted to the tip of growing complemented PT (left, arrow), while it accumulates inwards following the invaginated apical membrane in the over-expressing PT (right, arrows). Scale bar = 10 µm.

Time-lapse imaging of YFP fluorescence in growing PTs 6 hours after incubation showed that all PTs of the complemented line display the previously reported asymmetric distribution of YFP in the PM at the PT tip [19] and were growing normally (*n*>100, Figure 2A, left panels). In contrast, only 43% to 47% of ANX1 over-expressing PTs grew and exhibited the same YFP distribution (*n*>100 PTs, ANX1-OX #4 and #1, respectively). The remaining ANX1-OX PTs (53% to 57% of all PTs) had ceased to elongate and displayed PM invaginations at the PT tip as observed with both YFP and the lipophilic dye FM4-64 (Figure 2A, right panels). Intriguingly, in the ANX1 over-expressing PTs that had ceased to elongate, the PM at the tip kept growing inwards, creating tunnel-like structures, instead of outwards as normally observed for tip-growing cells (Figures 2B and S3A; Video S1). Invaginations can start early as they were observed even in pollen grains that did not yet produce a tube (Figure S3B). The PM invagination phenotype was also observed in ANX2 over-expressing lines, while we never saw it in any of the ANX1-YFP or ANX2-YFP complemented lines (*n*>100 PTs, two independent lines for each fusion protein). PM invaginations were accompanied by thick extracellular deposits of CW material (Figure 2A, asterisk), which were pectinaceous as revealed by Ruthenium red staining (Figure 2C). This finding indicates that secretion of CW material still occurred at the site of PM invagination. In addition, detailed observations of ANX-OX PTs showed that apical CW thickening occurred before the invagination of the apical membrane.

**Figure 3:**
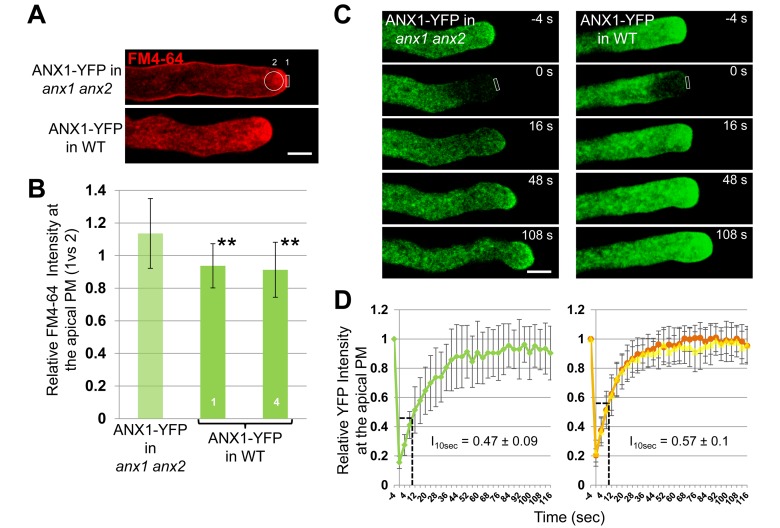
ANX RLK over-expressing pollen tubes do not exhibit endocytosis defects but display an increased rate of exocytosis. (A) Representative single median plane images of a normally growing PT of the ANX1-YFP complemented line (top) and a slow growing PT of the ANX1-YFP over-expressing line (bottom) treated for 5 min with FM4-64 (2 µM). FM4-64 derived fluorescence was quantified in the apical PM (region 1) and the apical cytoplasm (region 2) for *n*>25 PTs of each line. Note that there are more endocytotic and secretory vesicles in the apical cytoplasm of over-expressing PTs. See also corresponding Video S2 and (B). Scale bar = 5 µm. (B) Quantitative analysis of relative FM4-64 fluorescence in the apical PM *versus* the apical cytoplasm in growing PTs of one ANX1-YFP complemented and two over-expressing lines. Data are presented as mean values ± standard deviation (SD) (*n*>25 each). Double asterisks indicate significant differences from the complemented line according to a Student's *t* test with *p*<0.01. (C) Representative time-course imaging of FRAP for a complemented (left) and an over-expressing growing PT (right). Refer to Video S3 for more examples. Scale bar = 5 µm. (D) Quantitative analysis of FRAP time-courses of growing PTs of the complemented line (left, *n* = 18) and two over-expressing lines (right, *n*>17 for each). Relative intensity of apical PM-localized ANX1-YFP compared with fluorescence prior to photobleaching was used to quantify the rate of fluorescence recovery. FRAP signals are shown as mean values ± SD. The relative intensity after recovery for 10 s after photobleaching (I_10sec_) is indicated. See also corresponding Table S1.

Since the surface of both the PM and the secreted CW material increase at the tip, we hypothesize that the balance between endocytosis and exocytosis rates might be tilted towards exocytosis at the tip of ANX-OX PTs. This could be achieved by a decrease or increase in the rate of endocytosis or exocytosis, respectively, or by a combination of both. For example, CW accumulation and PM invaginations have also been reported for tobacco PTs that over-express the phosphatidylinositol-4-phosphate 5-kinases PIP5K4, PIP5K5, and PIP5K6 [29]–[31]. The PIP5K-OX phenotypes originate from an over-initiation of aborted endocytosis in PIP5K-OX PTs, which show a dramatic inhibition of FM4-64 uptake [30],[31]. To investigate this phenotype further, we conducted two types of experiments on growing PTs of complemented and ANX1-OX lines before they start to show PM invaginations and apical CW accumulation. First, we labeled PTs for 5 min with FM4-64, a styryl dye that quickly labels the PM and is internalized via endocytosis. In growing PTs of complemented lines (as in WT), FM4-64 is observed at the PM and in the apical cytoplasm as an inverted cone that presumably contains both endocytotic and secretory vesicles (Figure 3A and Video S2, upper panels). In growing ANX1-OX PTs, the same distribution was observed (Figure 3A and Video S2, lower panels), indicating that, in contrast to PIP5K-OX PTs, FM4-64 uptake and thus endocytosis was not impaired [30],[31]. However, FM4-64 fluorescence intensity at the PM *versus* the apical cytoplasm was significantly lower than in complemented PTs, suggesting that there were globally more endocytotic and secretory vesicles in ANX1 over-expressing PTs (Figure 3A and 3B; *n*>25 each, *p*<0.01).

As evidenced by Brefeldin A (BFA) treatment, a well-known inhibitor of exocytosis, ANX1-YFP is inserted at the apical PM via exocytosis (Figure S4A). Thus, we performed fluorescence recovery after photobleaching (FRAP) experiments for ANX1-OX and complemented PTs to analyze exocytosis dynamics in growing PTs as described previously [31],[32]. Photobleaching was applied to the tip of growing PTs and measurements of the recovery of YFP fluorescence in the apical PM of the PT tips were carried out every 4 seconds. For ANX1 complemented PTs, the relative fluorescence recovery in the PM 10 seconds after photobleaching (I_10sec_) reached on average 47%±9% of the maximum relative fluorescence with a PT growth rate of 4.02±1.41 µm min^−1^ (*n* = 18; Figure 3C and 3D, left panels; Table S1; Video S3). No correlation was observed between I_10sec_ and the fluorescence intensity pre-bleaching (R^2^ = 0.0092; Figure S4B), suggesting that the secretion rate of new ANX1-YFP fusion protein in the PM is independent of the amount of fusion protein originally present in the PM. Furthermore, no correlation was observed between I_10sec_ and PT growth rate (R^2^ = 0.0001; Figure S4C), indicating that exocytosis and PT growth rate do not share a direct linear relationship.

Interestingly, for PTs of both ANX1 over-expressing lines, I_10sec_ was significantly higher than in the complemented line (57%±10%, *p*<0.01 for line #1; 57%±13%, *p*<0.05 for line #4), while their PT growth rate was significantly decreased to 1.34±0.68 µm min^−1^ and 1.78±0.53 µm min^−1^, respectively (*n* = 17 and *n* = 20 for ANX1-OX lines #1 and #4, respectively, *p*<0.01; Figure 3C and 3D right panels; Table S1; Video S3). The faster fluorescence recovery is unlikely to be due to a secondary effect of slow PT growth, because all the mutant PTs tested so far in FRAP experiments, namely DN-ROP1-OX (dominant negative ROP1), CA-ROP1-OX (constitutively active ROP1), RIC3-OX, RIC4-OX, and PIP5K6-OX grow slower than controls and show an inhibition of fluorescence recovery [31],[32]. Thus, increased rates of fluorescence recovery at the apical PM indicate that the rate of exocytosis is increased at the apical PM of growing ANX1-OX PTs as compared to controls.

Altogether, our results support the hypothesis that ANX over-expression tilts the balance of exo- to endocytosis towards more exocytosis, which progressively leads to CW accumulation. PT growth slows down as the apical CW thickness increases. When the latter reaches a certain threshold where the CW is not deformable anymore, expansion ceases and apical PM grows inwards due to continuing exocytosis.

### Disruption of the Pollen-Expressed NADPH Oxidases RbohH and RbohJ Triggers Anxur-Like Phenotypes

**Figure 4:**
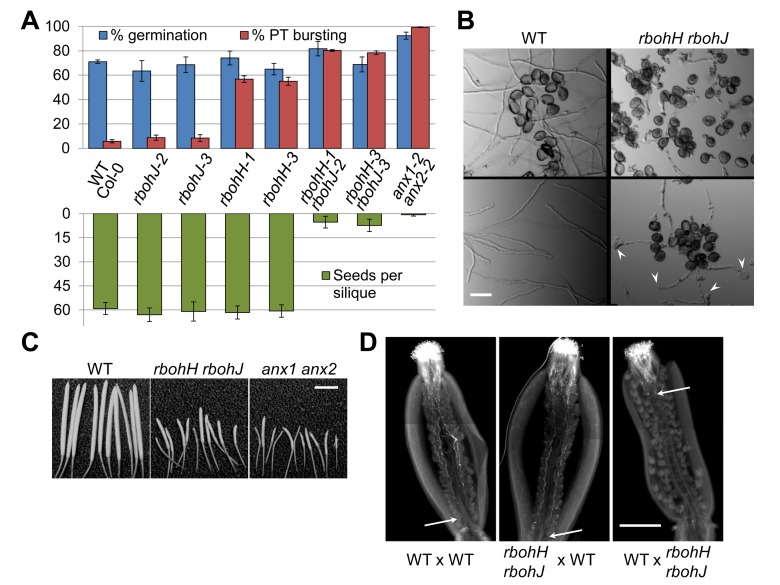
*rbohH rbohJ* mutant pollen display *anxur*-like phenotypes. (A) Quantification of pollen germination and PT rupture percentages (top histogram) and seed per siliques (bottom histogram) for WT, single, and double *rboh* as well as *anx1 anx2* mutant plants. Data are mean ± standard error of the mean (SEM) of three independent experiments with more than 150 pollen grains or ten siliques per genotype and experiment. (B) Representative overview images of WT and *rbohH rbohJ* pollen grown *in vitro* for 5 h. Up to 80% of germinated pollen from *rbohH rbohJ* ruptured with clear traces of cytoplasmic content that was released into the medium (top right), while the remaining germinated grains produce PTs that will burst later on (bottom right, arrowheads) as opposed to WT PTs that grow normally (bottom left). Scale bar = 50 µm. (C) Photographs of siliques from WT, *rbohH rbohJ*, and *anx1 anx2* plants. Scale bar = 500 µm. (D) Representative images of aniline blue staining of a WT pistil pollinated with WT pollen (left), a *rbohH rbohJ* pistil with WT pollen (middle), and a WT pistil with *rbohH rbohJ* pollen (right). Eighteen hours after manual pollination, WT PTs (left and middle panels) had grown through the entire pistil to reach the female gametophytes. In contrast, most of the *rbohH rbohJ* mutant PTs (right) were arrested in the transmitting tract. White arrows indicate the tip of the longest PT. Scale bar = 5 mm.

A better understanding of how the ANX RLKs regulate exocytosis requires the identification of downstream components of the ANX-dependent pathway. Recently, FER, which is the closest homologue of the ANX RLKs in *Arabidopsis*, has been shown to function as an upstream regulator of the ROP2/NADPH oxidase RbohC signaling pathway that controls ROS-dependent root hair growth [18]. Moreover, down-regulation of a pollen-expressed NADPH oxidase and application of ROS scavengers inhibit PT growth in tobacco [21]. Thus, we hypothesized that pollen-expressed NADPH oxidases could be downstream components of the ANX RLK pathway that coordinates CW integrity and PT growth. In *Arabidopsis*, NADPH oxidases belong to a family with ten members, two of which, RbohH (At5g60010) and RbohJ (At3g45810), sharing 81% amino acid identity, define a subgroup that is preferentially expressed in pollen (Figure S5C) [13],[14]. We isolated two independent single T-DNA insertional mutants for each of these NADPH oxidases, namely *rbohH-1* (GABI_028G04), *rbohH-3* (SALK_136917), *rbohJ-2* (SAIL_31_D07), and *rbohJ-3* (SALK_050665), which show little or no expression of the corresponding gene (Figure S5B). Pollen germination assays showed that PTs of single *rbohJ-2* and *rbohJ-3* mutant plants behaved like WT (∼8.5% bursting), while around 57% of PTs of single *rbohH-1* and *rbohH-3* mutant plants ruptured *in vitro* (Figure 4A). However, this mild PT rupture phenotype did not significantly reduce seed set or TE_M_ *in vivo* (Figure 4A; Table S2). To investigate whether *RbohH* and *RbohJ* are redundant, single mutants were crossed to generate independent double mutant *rbohH-1 rbohJ-2* and *rbohH-3 rbohJ-3* plants. First, double homozygous mutant plants were only rarely found in the progeny of *rbohH-1/RbohH rbohJ-2/rbohJ-2* and *rbohH-3/RbohH rbohJ-3/rbohJ-3* (Table S3). Secondly, PTs of both independent *rbohH-1 rbohJ-2* and *rbohH-3 rbohJ-3* double mutants ruptured up to 80% *in vitro* (Figure 4A and 4B). The remaining germinating grains produced longer PTs, but they eventually also burst (Figure 4B). As a consequence, double homozygotes for *rbohH-1 rbohJ-2* and *rbohH-3 rbohJ-3* were partially sterile, producing only five to seven seeds per silique as compared to ∼60 seeds in WT or single mutant plants (Figure 4A and 4C). As evidenced by aniline blue staining after reciprocal crosses of *rbohH rbohJ* with WT, this sterility was due to the double mutant pollen being unable to grow sufficiently *in vivo* to reach and fertilize the female gametophytes (Figure 4D). This was further supported by analyses of male and female transmission efficiencies (TEs) of the *rbohH-1 rbohJ-2* and *rbohH-3 rbohJ-3* mutations, which showed a greatly reduced TE_M_ while TE_F_ was not significantly affected (Table S2).

Taken together, these results provide compelling evidence that disruption of both *RbohH* and *RbohJ* leads to spontaneous PT rupture, preventing PTs to reach and fertilize the female gametophytes *in vivo*. Interestingly, all the above mentioned phenotypes are reminiscent of the *anx1 anx2* double mutant phenotype [19],[20]. Moreover, our results show partial functional redundancy between pollen-expressed NADPH oxidases, with *RbohH* being able to perfectly substitute for the loss of *RbohJ*, while the latter can only partially substitute for the loss of *RbohH*.

### ANX1 Over-expression Phenotypes Depend on Functional RbohH and RbohJ NADPH Oxidases

**Figure 5:**
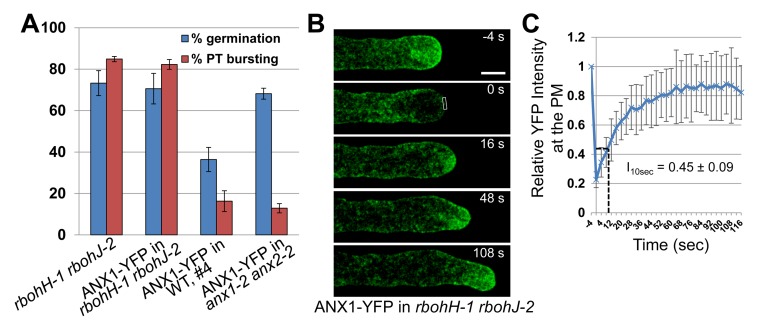
ANX1-YFP over-expression phenotypes are dependent on functional *RbohH* and *RbohJ*. (A) Quantification of pollen germination and PT rupture for *rbohH-1 rbohJ-2*, ANX1-YFP in WT (line #4), ANX1-YFP in *anx1-2 anx2-2* (complemented line), and ANX1-YFP in *rbohH-1 rbohJ-2* plants. Data are mean ± standard error of the mean (SEM) of three independent experiments with more than 150 pollen grains per genotype and experiment. (B) Representative time-course imaging of FRAP for a *rbohH-1 rbohJ-2* PT expressing ANX1-YFP. Scale bar = 5 µm. (C) Quantitative analysis of FRAP time-courses for growing PTs of ANX1-YFP in *rbohH-1 rbohJ-2* (*n* = 24). Relative intensity of apical PM-localized ANX1-YFP compared with fluorescence prior to photobleaching was used to quantify the rate of fluorescence recovery. FRAP signals are shown as mean values ± standard deviation (SD). The relative intensity after recovery for 10 s after photobleaching (I_10sec_) is indicated. See also corresponding Table S1.

To test whether *RbohH* and *RbohJ* are indeed downstream effectors of the ANX RLK-dependent pathway, the strong ANX1-OX line (#4) was crossed to *rbohH-1 rbohJ-2* double mutant plants. Partially male sterile plants homozygous for *rbohH-1 rbohJ-2* and homozygous for ANX1-YFP were retrieved in the F2 generation. Intriguingly, *rbohH-1 rbohJ-2* pollen strongly expressing ANX1-YFP behaved exactly like *rbohH-1 rbohJ-2* pollen with germination and PT rupture rates of ∼70% and 82%, respectively (Figure 5A). Furthermore, none of the growing PTs (*n*>100) of *rbohH-1 rbohJ-2* plants homozygous for ANX1-YFP displayed CW accumulation or PM invagination, phenotypes observed in ANX1-OX PTs. To independently confirm these results, we directly transformed *rbohH-1 rbohJ-2* mutant with ANX1-YFP fusion. Four independent, partially male sterile *rbohH-1 rbohJ-2* transgenic lines homozygous for ANX1-YFP were recovered in the T2 generation. Again, neither CW accumulation nor PM invagination was observed in growing PTs (*n*>100 PTs for each), which eventually ruptured similar to the *rbohH-1 rbohJ-2* PTs without the ANX1-OX construct. Furthermore, FRAP analyses also showed that the fast recovery rate at the apical PM observed in ANX1-OX PTs was suppressed in the *rbohH rbohJ* background as I_10sec_ for *rbohH rbohJ* PTs over-expressing ANX1-YFP was similar to the complemented line (45%±9%, *n* = 23; Figure 5B and 5C; Table S1). Interestingly, a few *rbohH rbohJ* PTs over-expressing ANX1-YFP did not recover 80% of the initial fluorescence, a phenomenon that was never observed in controls or ANX1-YFP over-expressing PTs (Figure S6), indicating that exocytosis may become defective in these *rbohH rbohJ* ANX1-OX PTs.

In summary, these results demonstrate that ANX1-OX phenotypes are dependent on functional *RbohH* and *RbohJ* and, consequently, that these pollen-expressed NADPH oxidases are positive downstream effectors of the ANX RLK-dependent pathway.

### NADPH Oxidases RbohH and RbohJ Are Responsible for H_2_O_2_ Production at the Tip of Growing Pollen Tubes

**Figure 6:**
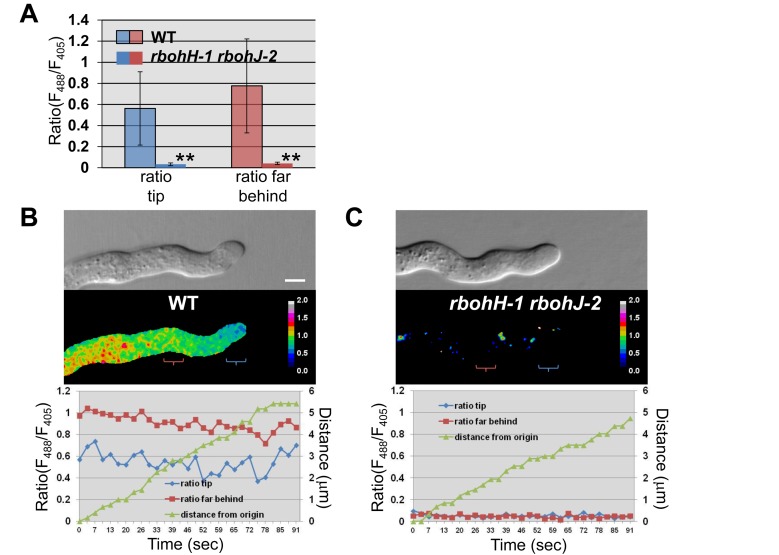
H_2_O_2_-sensitive HyPer sensor ratiometric imaging shows that RbohH and RbohJ are responsible for H_2_O_2_ production at the tip of growing pollen tubes. (A) Quantification of HyPer ratio (F_488_/F_405_) at the tip and further back in the shank of growing WT (*n* = 27) and *rbohH-1 rbohJ-2* (*n* = 22) PTs. Data are shown as the mean of ratios over 90 seconds±standard deviation (SD). Double asterisks indicate significant differences from the WT according to a Student's *t* test with *p*<0.01. (B) Representative images of a growing WT PT expressing cytosolic HyPer and the corresponding histogram displaying the ratios (F_488_/F_405_) at the tip (blue line) and behind the tip (red line) over 90 s, as well as the travelled distance of the PT tip (green line). The blue and red parentheses indicate where the circles of 4 µm diameter were positioned for measurements at the tip and behind the tip, respectively. See corresponding Video S4. Scale bar = 5 µm. (C) Representative images of growing *rbohH-1 rbohJ-2* PT expressing cytosolic HyPer. See (B) for details.

To check whether disruption of *RbohH* and *RbohJ* impairs the production of ROS, we made use of the fluorescent ROS-sensitive dye 5-(and 6-)chloromethyl-2′,7′-dichlorodihydrofluorescein diacetate (CM-H_2_DCFDA) to stain PTs of WT and *rbohH rbohJ* double mutants. Fluorescence quantification of the apical cytoplasm in growing PTs treated for 5 minutes with 2 µM CM-H_2_DCFDA showed that PTs of the *rbohH-1 rbohJ-2* and *rbohH-3 rbohJ-3* double mutants displayed only 25% of the CM-H_2_DCFDA-derived fluorescence signal observed in WT PTs (*p*<0.01; Figure S7A and S7C). These low levels of CM-H_2_DCFDA-derived fluorescence were not due to a defect in dye uptake, as mutant and WT PTs exhibited the same level of fluorescence derived from the ROS-insensitive dye fluorescein diacetate (FDA) (Figure S7B and S7D). These results show that ROS production is indeed impaired in *rbohH rbohJ* PTs as expected for NADPH oxidase mutants [12]. However, because CM-H_2_DCFDA oxidation is sensitive to different reactive oxygen and nitrogen species, sensitive to light, and irreversible, this dye cannot be used to monitor ROS production over time in growing PTs. Thus, we generated stably transformed *Arabidopsis* lines with PT expression of the genetically encoded YFP-based ratiometric sensor HyPer, which has been shown to faithfully report H_2_O_2_ production in bacteria, animal, and plant cells [33],[34]. Curiously, in growing WT PTs expressing cytosolic HyPer (*n* = 27), the HyPer activity measured as the ratio of F_488_/F_405_ was stronger in the shank of PTs than at the tip (Figure 6A and 6B). We hypothesized that this strong shank activity could either be due to the presence of H_2_O_2_-producing organelles, such as mitochondria and/or peroxisomes in this region, an artifact of HyPer due to its pH sensitivity, or a combination of both. Indeed, it was shown that HyPer's activity artificially increases when the pH increases [33] and that PTs display a pH gradient with an acidic tip and a alkaline shank [35]. Interestingly, at the tip of growing PTs, HyPer activity displayed irregular oscillations originating from the tip periphery (Figures 6B and S8A; Video S4). However, oscillations of HyPer activity did not seem to correlate with growth rates (Figure S8B). In growing *rbohH-1 rbohJ-2* PTs, HyPer activity was 16 and 18 times lower at the tip and in the shank, respectively, as compared to the WT (*n* = 22, *p*<0.001; Figure 6A and 6C). This indicates that membrane-bound RbohH and RbohJ are responsible for most of the H_2_O_2_ production revealed by the HyPer sensor. Moreover, since HyPer activity in the shank was also strongly reduced in *rbohH-1 rbohJ-2* double mutant PTs (Figure 6A and 6C), the strong activity in the shank of WT PTs is likely due to propagation of the tip-derived H_2_O_2_ in the alkaline shanks, which artificially increases HyPer activity.

To investigate if Rboh localization is consistent with the Rboh-dependent H_2_O_2_ production observed at the tip-periphery, we transformed partially sterile *rbohH-3 rbohJ-3* plants with a green fluorescent protein (GFP)-RbohH fusion. Forty-four independent T1 transgenic lines out of 50 displayed rescue of sterility with WT-like elongated siliques (e.g., for three independent T1 lines with good GFP expression, the average of seeds per silique was 40.8±3.8, 40.9±11.1, and 43.4±6.9 as opposed to 6.2±3.5 in untransformed *rbohH-3 rbohJ-3*, *n* = 12 siliques per plant). *In vitro* pollen growth assays confirmed that the *rbohH rbohJ* bursting phenotypes were rescued by GFP-RbohH (Figure S8C) and that GFP-RbohH localized polarly to the plasma membrane at the tip of growing PTs (Figure S8D, left panels). These results show that GFP-RbohH protein fusion is functional and that its localization is consistent with both ANX1-YFP localization (Figure 2A, left panels) and Rboh-dependent H_2_O_2_ production at the tip periphery (Figure S8A). Furthermore, unlike the *rbohH rbohJ* complemented plants, in WT plants expressing the GFP-RbohH fusion, PM invagination and over-accumulation of CW material were also observed (Figure S8D, right panels), although these phenotypes appeared milder and less frequent than in ANX1-OX PTs.

### Calcium Homeostasis Is Impaired in Growing *rbohH rbohJ* Double Mutant Pollen Tubes

ROS and H_2_O_2_ have been shown to regulate calcium-permeable channels, e.g., in protoplast of root hairs [12] and pear pollen [36], and a tip-focused Ca^2+^ gradient is essential for polar growth [37]. Therefore, we crossed WT plants expressing the genetically encoded FRET-based Ca^2+^-cameleon YC3.60 in PTs [38] with the *anx1-2 anx2-2* and *rbohH-1 rbohJ-2* double mutants, and partially male sterile *anx1-2 anx2-2* and *rbohH-1 rbohJ-2* plants homozygous for YC3.60 were recovered in subsequent generations. Cytosolic Ca^2+^ concentrations ([Ca^2+^]_cyt_, measured as F_CFP_/F_Venus_) were monitored over time at the PT tip and behind the tip when possible, and compared to YC3.60-expressing WT PTs grown and imaged under the same conditions. First, we attempted to study Ca^2+^ dynamics in *anx1 anx2* bulges before bursting, young growing WT PTs, and arrested WT bulges. Bulges of *anx1 anx2* never produced a growing tube, and only two out 17 burst during imaging. Interestingly, for both *anx1 anx2* bulges that eventually burst, a sudden increase of [Ca^2+^]_cyt_ was observed (Figure S9A, white arrow) before the first visible sign of rupture (Figure S9A, black arrow; Video S5). However, before bursting, [Ca^2+^]_cyt_ in non-growing *anx1 anx2* bulges was on average lower than at the tip of growing WT PTs but similar to the arrested WT bulges (Figure S9B). Because one cannot conclude if the decreased levels of [Ca^2+^]_cyt_ in *anx1 anx2* are due to the lack of ANX RLKs or rather to an indirect effect of arrested growth, we focused on studying Ca^2+^ dynamics in growing WT PTs and *rbohH rbohJ* pollen grains, which produce a few growing PTs that eventually burst.

**Figure 7:**
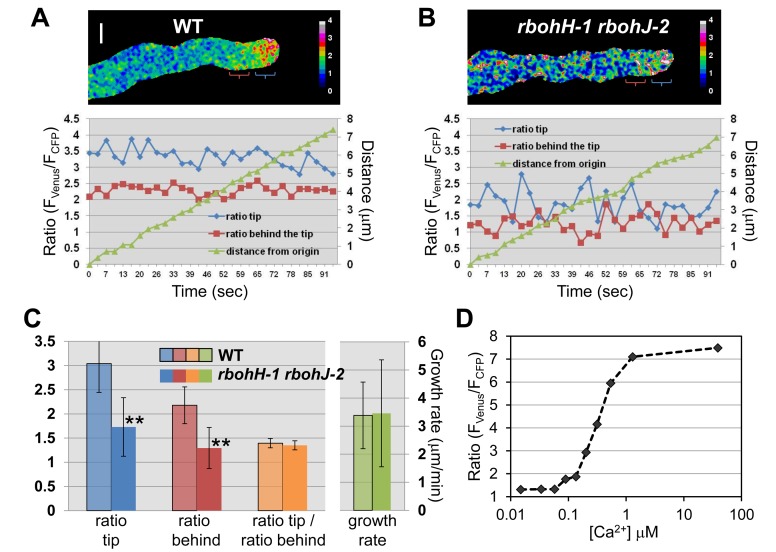
Ca^2+^-sensitive cameleon YC3.60 ratiometric imaging shows that [Ca^2+^]_cyt_ levels are decreased and less steady in growing *rbohH-1 rbohJ-2* pollen tubes. (A) Representative images of growing WT PTs expressing cytosolic YC3.60 and the corresponding histogram displaying the ratios (F_CFP_/F_Venus_) at the PT tip (blue line) and behind the tip (red line) over 90 seconds, as well as the travelled distance of the PT tip (green line). On the ratiometric image, the blue and red parentheses indicate where the circles of 4 µm diameter were positioned for measurements at the PT tip and behind the tip, respectively. See also corresponding Video S6. Scale bar = 5 µm. (B) Representative images of growing *rbohH-1 rbohJ-2* PTs expressing cytosolic YC3.60. For details, see (A). (C) Quantification of YC3.60 ratio (F_CFP_/F_Venus_) at the PT tip (blue bars) and just behind the tip (red bars), as well as the tip-focused Ca^2+^gradient (ratio at the tip/ratio behind the tip; orange bars) and growth rates (green bars) of growing WT (*n* = 46) and *rbohH-1 rbohJ-2* (*n* = 30) PTs. Data are shown as the mean of ratios or growth rates over 90 seconds ±standard deviation (SD). Double asterisks indicate significant differences from the WT according to a Student's *t* test with *p*<0.01. (D) Titration curve for YC3.60.

In steadily growing WT PTs, the tip-focused Ca^2+^ gradient (i.e., higher [Ca^2+^]_cyt_ at the PT tip compared to behind the tip) was always observed and quite stable (*n* = 46; Figures 7A, 7C, S10A, and S10D; Video S6). Furthermore, as reported previously for *in vitro* grown *Arabidopsis* PTs [38],[39], but unlike lily PTs [40], we did not observe regular oscillations for either the PT growth rate or [Ca^2+^]_cyt_ (Figure 7A). In growing *rbohH rbohJ* PTs (*n* = 30), [Ca^2+^]_cyt_ was significantly lower than in the WT (*p*<0.001 for both tip and behind the tip regions; Figure 7B and 7C; Video S6). However, the tip-focused Ca^2+^ gradient and the PT growth rate were on average similar to that of WT PTs (Figure 7B and 7C; *p* = 0.054 for gradient, *p* = 0.84 for growth rate). But both the tip-focused Ca^2+^ gradient and the PT growth rate were significantly less steady over time in the *rbohH rbohJ* double mutant than in the WT, as evidenced by a significantly higher variance (*p* = 4.1280•10^−13^ and *p* = 0.008737 for [Ca^2+^]_tip_/[Ca^2+^]_behind_ and growth rate, respectively; Figure S10A–S10F). The steady and jerky growth rate of WT and *rbohH rbohJ* PTs, respectively, were quite obvious during live-imaging of growing FM4-64 stained PTs (Video S7).

These results indicate that disruption of the pollen-expressed NADPH oxidases *RbohH* and *RbohJ* does not abolish the tip-focused Ca^2+^ gradient but results in PTs that display (i) overall lower [Ca^2+^]_cyt_ levels, and (ii) unstable tip-focused Ca^2+^ gradients and growth rates. Finally, increasing the external [Ca^2+^] in the germination medium from 5 mM to 15 mM or 30 mM, significantly decreased the rupture of *rbohH rbohJ* PTs, while lowering the external [Ca^2+^] had the opposite effect (*p*<0.05; Figure S11). These findings indicate that supplementing Ca^2+^ externally can partially stabilize the growth of *rbohH rbohJ* PTs. Conversely, decreasing external [Ca^2+^] strongly, increases the frequency of PTs that rupture in the WT (Figure S11), consistent with a pioneering report from the 1980s [41].

## Discussion

### The Pollen-Expressed NADPH Oxidases RbohH and RbohJ Are Downstream Components of the ANX RLK-Dependent Cell Wall Integrity Pathway

In tip-growing root hairs, *FER* and *RbohC/RHD2* have been proposed to function in the same pathway based on the facts that: (i) *fer* and *rbohC/rhd2* display similar phenotypes with stunted, collapsed, and bursting root hairs, and (ii) that roots and root hairs of *fer* and one FER-OX line accumulate less and more ROS than WT, respectively [12],[18]. Similarly, we show here that two independent *rbohH rbohJ* double mutants display *anx*-like phenotypes, i.e., PTs that burst, preventing them from growing to fertilize the female gametophytes (Figure 4). Consequently, both *anx1 anx2* and *rbohH rbohJ* mutant plants are nearly male sterile. In addition, over-expression of both ANX1-YFP and GFP-RbohH triggers over-accumulation of membrane and CW materials (Figures 2 and S8D, right panels). Furthermore, we provide strong genetic evidence for the NADPH oxidases to function downstream of the ANX RLKs, by showing that the phenotypes observed in ANX-OX lines are abolished in the *rbohH rbohJ* mutant background (Figure 5). Therefore, the CrRLK1L-NADPH oxidase signaling module appears to be conserved in tip-growing cells. However, it is unlikely that the CW integrity pathway in pollen is a carbon copy of the root hair pathway, as the biological functions, growth habits and patterns, CW compositions, and growth environments are quite different between these tip-growing cells [42]. For example, in root hairs *FER* has been shown to positively regulate RbohC-dependent ROS production through ROP2-signaling [18]. In PTs, however, it remains unclear whether ANX RLKs also activate the NADPH oxidases RbohH and RbohJ through ROP-signaling, because over-activation of ROP-signaling leads to growth depolarization but does not trigger CW accumulation, PM invagination, or increased apical exocytosis [32],[43],[44], as we observed it in ANX1-OX lines (Figures 2 and 3).

### NADPH Oxidases RbohH and RbohJ Are Responsible for ROS and Pulsating H_2_O_2_ Production at the Tip of Growing Pollen Tubes

Our understanding of the role of NADPH oxidase-derived ROS signaling in plant development and in responses to abiotic and biotic stresses has improved tremendously over the past few years [13],[14],[27]. Production of different ROS species has been imaged in different plant tissue and cell types, but because of the irreversible oxidation of the different dyes used (e.g., diaminobenzidine tetrahydrochloride, nitro blue tetrazolium [NBT], H_2_DCFDA and derivatives) meaningful information about the dynamics of ROS production is still scarce [45]. GFP-based, genetically encoded sensors such as roGFPs and HyPer, which display reversible changes in fluorescence to alterations in redox/ROS levels, have been successfully developed and tested in plant cells [45]. However, none of them have been assayed in a mutant background affecting ROS-producing enzymes. Here, we used the cytoplasmic H_2_O_2_-selective HyPer sensor expressed in PTs in a *rbohH rbohJ* NADPH oxidase-deficient mutant background to gain more insights into H_2_O_2_ production in tip-growing cells. HyPer activity displayed irregular oscillations at the tip of growing WT PTs (Figures 6B and S8). HyPer oscillations are unlikely due to pH oscillations reported for the tip of growing PTs, because (i) pH at the tip varies [35] in a range where HyPer is not really pH-sensitive [33], and (ii) HyPer activity is completely abolished in growing *rbohH rbohJ* mutant PTs (Figure 6A and 6C). Moreover, HyPer activity originated from the periphery of the growing tip (Figure S8A), which is consistent with (i) the tip-preferential PM RbohH localization (Figure S8D, left panels) and the reported PM localization of other NADPH oxidases [9],[46], (ii) the NADPH oxidase activity reported at the PM [47],[48], and (iii) the extracellular, tip-localized O_2_^.^^−^ distribution revealed by NBT staining of PTs [21],[48].

### NADPH Oxidases Fine Tune Calcium Homeostasis

The exact mechanism by which NADPH oxidase-dependent ROS regulate polar growth is still not fully understood. One reason for this is that quantitative information with good temporal and spatial resolution is difficult to obtain from growing CrRLK1L and/or NADPH-oxidase mutant cells (root hairs or PTs), owing to their rapid loss of cellular integrity. On one hand, the NADPH oxidase RbohC has been proposed to generate ROS that activate Ca^2+^-permeable channels at the PM to establish the tip-focused Ca^2+^ gradient and to promote expansion at the tip of root hairs [12],[25]. On the other hand, a tip-focused Ca^2+^ gradient was observed in *rbohC* root hairs under certain conditions, indicating that *RbohC* was not essential to generate the Ca^2+^ gradient, but rather plays a role in restricting growth to the tip by rigidifying the CW behind the tip [26]. On the basis of our results we propose a third alternative. Unlike *anx1 anx2*, a small but appreciable number (∼20%) of germinating *rbohH rbohJ* pollen grains are able to produce longer tubes *in vitro* that, however, will eventually burst, too. We took advantage of this opportunity to study [Ca^2+^]_cyt_ dynamics with a good spatial and temporal resolution on growing NADPH oxidase-deficient PTs. First, the tip-focused Ca^2+^ gradient, visualized by the ratio between [Ca^2+^]_tip_/[Ca^2+^]_behind_, was clearly visible in growing *rbohH rbohJ* PTs, confirming that NADPH oxidases are not required to generate the Ca^2+^ gradient. However, unlike steadily growing WT PTs, which maintain a constant Ca^2+^ gradient over time (Figure S10A, S10D, and S10E), *rbohH rbohJ* PTs displayed a very unstable gradient, which could sometimes be steep but was abolished a few seconds later (Figure S10B, S10D, and S10E). This was correlated with more variable growth rates in *rbohH rbohJ* mutant compared to steadily growing WT PTs. Moreover, the global cytosolic Ca^2+^ levels were significantly lower in the growing *rbohH rbohJ* mutant PTs compared to WT PTs (Figure 7C). An increase in external [Ca^2+^] partially rescued the rupture of *rbohH rbohJ* PTs, while lowering the external [Ca^2+^] increased PT rupture in both the mutant and WT (Figure S11). This is in agreement with previous studies, which showed that lowering external [Ca^2+^] or limiting/blocking Ca^2+^ influx causes PTs and root hairs to burst [41],[49]. The data reported here are consistent with NADPH oxidase-dependent ROS activating Ca^2+^-permeable channels for Ca^2+^ influx [12],[36]. However, we propose that these yet unidentified channels do not generate the tip-focused Ca^2+^ gradient on their own; rather, they fine tune the Ca^2+^ gradient by stabilizing it to sustain steady growth of *Arabidopsis* PTs. It is noteworthy that different types of PM-localized Ca^2+^ channels have been characterized recently in tip-growing cells [37],[50]. Among these, the Cyclic-Nucleotide-Gated Channel (CNGC) family is of particular interest, because single *cngc18* or double *cngc7 cngc8* mutant PTs spontaneously rupture after germination or produce kinky PTs that often burst as well [51],[52]. Thus, CNGCs constitute good candidates for Ca^2+^ channels that are regulated by the CrRLK1L-NADPH oxidase signaling module at the PM. Annexins are also possible candidates as ANN1 has recently been shown to function as a ROS-activating Ca^2+^ transporter in root cells [53].

### ANX RLKs Regulate Exocytosis at the Apical Plasma Membrane of Pollen Tubes

One of the many roles proposed for the tip-focused Ca^2+^ gradient is to facilitate and stimulate exocytosis at the site of growth [54],[55], where the exocyst complex has been shown to function [56]. Increasing external [Ca^2+^] leads to root hair and PT growth inhibition and CW thickening [41],[49]. However, in this case it is not clear whether the accumulation of secreted CW material is due to an increase of the exocytosis rate or to uncoupling of exocytosis (that otherwise remains the same) from growth. Interestingly, we found that ANX over-expressing PTs grow slower than controls and also display CW accumulation (Figure 2). By performing FRAP analyses in the apical membrane of growing PTs of WT, *anx1 anx2*, and *rbohH rbohJ* plants expressing the ANX1-YFP fusion protein, we show that the rate of exocytosis is significantly increased in ANX1-OX PTs compared to controls. In contrast, for some of the *rbohH rbohJ* mutant PTs that have low calcium levels and an unsteady Ca^2+^ gradient, the recovery was impaired during our analysis. In agreement, the Ca^2+^channel blocker LaCl_3_, which has been shown to trigger the rupture of root hairs [49], appears to inhibit FRAP at the PT tip [32].

### A Model for the CrRLK1L-NADPH Oxidase Signaling Pathway

Altogether our data are consistent with the following sequence of events: ANX RLKs positively regulate the NADPH oxidases RbohH and RbohJ, possibly through ROP signaling, to periodically produce ROS. Subsequently, ROS activate Ca^2+^-permeable channels for calcium influx to fine tune the tip-focused Ca^2+^ gradient, which in turn sustains secretion at the apical tip enabling PTs to elongate steadily without a loss of CW integrity. Perturbations of the pathway by over-expressing ANX RLKs would lead to a NADPH oxidase-dependent over-production of ROS and Ca^2+^ influx at the PT tip, which in turn would increase the secretion rate of membrane and CW materials, progressively leading to growth cessation and membrane invagination. Conversely, disrupting the ANX RLKs or NADPH oxidase would abolish NADPH oxidases-dependent ROS production and impair the opening of ROS-activated Ca^2+^-permeable channels, thus limiting the cell's ability to buffer [Ca^2+^]_cyt_ variation that is required to maintain a steady tip-focused Ca^2+^ gradient. Consequently, the Ca^2+^ gradient and exocytosis at the PT tip would become erratic and, if not stabilized by compensatory mechanisms, the CW thickness would decrease until turgor pressure would lead to PT rupture. Finally, our model does not exclude that, in parallel to the signaling events described above, NADPH oxidase-dependent ROS and/or Ca^2+^ could directly alter CW properties, thereby affecting PT tip-growth. To investigate this possibility, direct measurements of the impact of ROS on CW properties during tip-growth would need to be established. We are confident that combining the continuously improving polar growth models and techniques to measure mechanical properties of growing cells [57],[58] with genetic approaches, will soon uncover some of the remaining mysteries of the fascinating coordination between CW integrity and polar growth.

## Materials and Methods

### Ruthenium Red, FM4-64, CM-H_2_DCFDA, and FDA Imaging

After 3 h to 5 h of *in vitro* incubation on solid germination medium, 100 µl of liquid germination medium containing 0.01% Ruthenium red (Sigma, R-2751) or 2 µM of either FM4-64 (Molecular Probes, Invitrogen, T3166), CM-H_2_DCFDA (Molecular Probes, Invitrogen, C6827), or FDA (Sigma, F7378) were applied for 5 min to PTs, then washed away with fresh dye-free medium before imaging. PTs stained with Ruthenium red were imaged with a Leica DM6000 (Leica Microsystems). PTs stained with either FM4-64, CM-H_2_DCFDA, or FDA were imaged with a Leica SP2 or SP5 confocal microscope. For FM4-64 stained PTs, the apical PM region was defined as the first 2.5 µm along PM at the apex. A circle (2.5 µm in diameter) 3 µm away from tip was chosen for measurement of apical cytosol intensity. Relative localization of the FM4-64 dye on the PM *versus* the apical cytosol was calculated to illustrate the degree of FM4-64 internalization. For CM-H_2_DCFDA and FDA stained PTs, a circle (4 µm in diameter) 3 µm away from tip was chosen to measure apical cytosol intensity. All dye-derived fluorescence intensities were measured using the ImageJ 1.47d software after background subtraction. PTs of different genotypes were all imaged and quantified under the same conditions.

### FRAP Imaging and Analyses

Growing PTs expressing ANX1-YFP in an *anx1-2 anx2-2* (complemented line), WT (ANX-OX, lines #1 and #4), and *rbohH-1 rbohJ-2* backgrounds were used for FRAP analyses with the same imaging and quantification parameters. The apical region of PTs was photobleached using 100% power of a 514-nm laser (Leica SP5), and the recovery of fluorescence was monitored every 4 s in the following 2 min. The apical PM region was defined on the bright-field pictures at every time frame as the first 2.5 µm along PM at the apex, and fluorescence intensities were measured with ImageJ 1.47d software after background subtraction. Relative intensity of PM-localized ANX1-YFP compared with fluorescence before photobleaching was used to quantify the speed of fluorescence recovery. See Table S1 for curve fitting.

### Ratiometric Imaging of HyPer and YC3.60 and Relative Analyses

Fluorescence in growing PTs of WT and *rbohH-1 rbohJ-2* expressing either HyPer or YC3.60 were acquired (Leica SP2 confocal microscope) and quantified (ImageJ 1.47d) in the exact same conditions. For HyPer, fluorescence was acquired with the sequential mode and excitation at 488 nm and emission between 500–540 nm for F_488_ and excitation at 405 nm and emission between 500–540 nm for F_405_. Two circular regions of interest (ROIs; 4 µm in diameter), one 0.5 µm, the other 20 µm away from the apex were drawn for measurement of apical cytosol and far behind the tip intensities, respectively, for each single time point of each PT. For YC3.60, excitation was 458 nm then emission 469–501 nm for F_CFP_ and 522–554 nm for F_Venus_. Two circular ROIs (4 µm in diameter), one 0.5 µm, the other 10 µm away from the apex, were drawn for measurement of apical cytosol and behind the tip intensities, respectively, for each single time point of each PT. All ratiometric measurements, i.e., F_488_/F_405_ and F_CFP_/F_Venus_, were determined with ImageJ 1.47d and its Ratio ROI Manager plugin after background subtraction. Ratiometric pictures were generated with the plugin Ratio Stack after median filtering. The YC3.60 titration curve (Figures 7D) was obtained as described before [38].

All primers used in this study are listed in Table S4. Additional protocols are described in Text S1.

## Supporting Information

Figure S1 **Over-expression of ANX RLKs inhibits pollen germination and increases pollen tube width.** (A) Quantification of pollen germination rate after 5 h of *in vitro* growth for WT, two and three independent ANX1-YFP and ANX2-YFP T3 complemented lines, respectively, as well as four independent T3 ANX1-YFP and ANX2-YFP over-expression lines. Data are representative of three experiments with more than 150 pollen for each genotype. The corresponding PT length measurements are displayed in Figure 1A. (B) Quantification of PT width after 5 h of *in vitro* growth for WT, one ANX1-YFP, and one ANX2-YFP T3 complemented lines, as well as three independent T3 ANX1-YFP and ANX2-YFP over-expression lines. Data represent mean values ± standard error of the mean (SEM) of three independent experiments with more than 40 PTs per genotype and experiment. Double asterisks indicate statistically significant differences from the WT according to a Student's *t* test with *p*<0.01.
(TIF)

Figure S2 **ANX1-YFP and ANX2-YFP fusion proteins complement the** ***anx1 anx2*** **pollen tube rupture phenotype.** Quantification of pollen germination rate and PT rupture after 5 h of *in vitro* growth for WT, *anx1-2 anx2-2*, *anx1-2/anx1-2 anx2-2/ANX2*, 6, and 5 independent T1 lines of ANX1-YFP in *anx1-2/anx1-2 anx2-2/ANX2* and ANX2-YFP in *anx1-1/anx1-1 anx2-1/ANX2*, respectively, as well as two and three independent T3 homozygous ANX1-YFP in *anx1-2 anx2-2* and ANX2-YFP in *anx1-1 anx2-1* complemented lines, respectively. More than 150 pollen were analyzed per genotype.
(TIF)

Figure S3 **ANX RLK over-expression triggers plasma membrane invagination.** (A) Median plane confocal image of an ANX1-YFP over-expressing PT, in which the apical membrane grows inwards. Filters are indicated on the left. Scale bar = 5 µm. (B) Single plane confocal image of plasma membrane invagination in an ANX1-YFP over-expressing pollen grain.
(TIF)

Figure S4 **BFA treatment disrupts the enrichment of ANX1-YFP at the apical plasma membrane.** (A) Representative median plane sections of *anx1 anx2* complemented PTs expressing ANX1-YFP with (right) or without (left) BFA treatment. Note that the YFP-derived fluorescence is much weaker in the apical membrane-derived region of interest of the BFA-treated PTs compared to that of non-treated PTs (left). The same regions of interest (ROIs) were used for FRAP experiments. No correlation between relative fluorescence recovery 10 s after photobleaching and original amount of fluorescent protein in the apical plasma membrane (B) nor with PT growth rate (C).
(TIF)

Figure S5 **Structure and expression of** ***Rboh*** **genes in** ***Arabidopsis*** **.** (A) The genomic organization of the pollen-expressed NADPH oxidase genes *RbohH* and *RbohJ* and positions of the *rbohH-1*, *rbohH-3*, *rbohJ-2*, and *rbohJ-3* T-DNA insertions. The orientation of the left border sequence of the respective T-DNAs is represented by black arrows. The positions of the primers used to genotype the mutants are indicated. (B) RT-PCR analyses of cDNAs from open-flowers show no *RbohH* transcripts in the T-DNA insertion lines *rbohH-1 and rbohH-3*. There are much less or no *RbohJ* transcripts in the T-DNA insertion lines *rbohJ-2* and *rbohJ-3*, respectively. *UBC21* (*At5g25760*) was used as a control. Amplification was performed for 30 cycles for *UBC21* and for 35 cycles for *RbohH* and *RbohJ*. (C) Multiple alignments of *Arabidopsis* Rboh proteins were performed with ClustalW 1.83 and the phylogenetic tree was reconstructed with MEGA4 using the protein sequence parsimony method (bootstrap test, 1,000 replicates). Black and grey circles at nodes indicate bootstrap values of more than 900 and between 800 and 900, respectively. The HsNOX5 was used as outgroup. The tree was then combined with the relative gene expression of *Arabidopsis* *Rboh* family members in various plant tissues according to the Genevestigator microarray database using the Meta-Profile Analysis tool, Anatomy Profile [59].
(TIF)

Figure S6 **Distribution of pollen tubes of ANX1-YFP in** ***anx1 anx2*** **, in wild-type (over-expressor line #1 and #4), and** ***rbohH rbohJ*** **backgrounds relative to the time required to recover 80% of the initial fluorescence.** Unlike PTs from complemented and over-expressor lines, some *rbohH rbohJ* PTs expressing ANX1-YFP were not able to recover 80% of the initial fluorescence at the apical plasma membrane.
(TIF)

Figure S7 ***rbohH rbohJ*** **pollen tubes display decreased levels of ROS-sensitive CM-H_2_DCFDA-derived fluorescence compared to the wild type.** (A) Single median plane images of growing WT and *rbohH rbohJ* PTs stained with the ROS-sensitive CM-H_2_DCFDA dye and imaged with the same settings. Scale bar = 5 µm. (B) Single median plane images of growing WT and *rbohH rbohJ* PTs stained with the ROS-insensitive FDA dye and imaged with the same settings. The scale is the same as in (A). (C) Quantification of ROS-sensitive CM-H_2_DCFDA-derived fluorescence in a circle with 4 µm diameter at the tip of growing WT and *rbohH rbohJ* PTs. Data are mean ± standard error of the mean (SEM) of three independent experiments with more than eight PTs per genotype and experiment. Double asterisks indicate significant differences from the WT according to a Student's *t* test with *p*<0.01. (D) Quantification of ROS-insensitive FDA-derived fluorescence in a circle with 4 µm diameter at the tip of growing WT and *rbohH rbohJ* PTs. Data are mean ± SEM of three independent experiments with more than eight PTs per genotype and experiment.
(TIF)

Figure S8 **Pulsating H_2_O_2_-sensitive HyPer activity at the tip of growing wild-type pollen tubes correlates with GFP-RbohH localization.** (A) Time-course ratiometric imaging of the tip of growing WT PT expressing cytosolic HyPer and the corresponding histogram (B) displaying the ratio (F_488_/F_405_) at the tip (blue line) over 90 s, as well as the PT growth rates (green line). Scale bar = 5 µm. (C) *In vitro* PT growth assay showing that the GFP-RbohH protein fusion complements the *rbohH rbohJ* PT bursting phenotype in T1 *rbohH-3 rbohJ-3* heterozygous for GFP-RbohH. Left, *rbohH-3 rbohJ-3* pollen. Right, pollen of a representative T1 *rbohH-3 rbohJ-3* line expressing GFP-RbohH. (D) Representative single median plane images of a normally growing PT of a GFP-RbohH complemented line (left) and an arrested PT of an GFP-RbohH over-expressing line with apical membrane invagination (right) and over-accumulation of CW material (asterisk). The different filters are indicated. Before imaging, PTs were treated for 5 min with germination liquid medium containing FM4-64 (2 µM). Scale bar = 5 µm.
(TIF)

Figure S9 **Ca^2+^− sensitive cameleon YC3.60 ratiometric imaging shows that [Ca^2+^]_cyt_ levels are decreased in** ***anx1 anx2*** **bulges compared to young, growing wild-type pollen tubes but are similar to arrested wild-type bulges.** (A) Representative ratiometric images of young growing WT PT, arrested WT bulge, and bursting *anx1 anx2* bulge expressing cytosolic YC3.60. On the ratiometric images, the black circles represent the region of interests of 4 µm diameter used for measurements at the PT tip. At time = 76 s, a white arrow indicates a sudden increase of [Ca^2+^]_tip_ in *anx1 anx2* before the bulge actually bursts (black arrow at time = 84 s). Note how external Ca^2+^ enters the *anx1 anx2* bulge once it has ruptured. See also corresponding Video S5. The calibration bar is the same as in Figure 7A. Scale bar = 5 µm. (B) Quantification of YC3.60 ratio (F_CFP_/F_Venus_) at the tip of young, growing WT PTs, arrested WT bulges, and *anx1 anx2* bulges before rupture (*n*>15 for each category). Data are shown as the mean of ratios over 90 s±standard deviation (SD). Double asterisks indicate significant differences from the growing WT PTs according to a Student's *t* test with *p*<0.01.
(TIF)

Figure S10 **Tip-focused Ca^2+^ gradient and pollen tube growth rates are less stable in** ***rbohH-1 rbohJ-2*** **than in the wild type.** Representative images of growing WT (A) and *rbohH-1 rbohJ-2* (B) PTs expressing cytosolic YC3.60 and the corresponding histograms displaying the ratio of [Ca^2+^]_tip_/[Ca^2+^]_behind_ (i.e., tip-focused Ca^2+^ gradient, orange line) over 90 s, and the travelled distance of the PT tip (green line). Note how both the tip-focused Ca^2+^ gradient and the PT growth rate are more stable in WT compared to the mutant. Scale bar = 5 µm. (C) Histogram of the variance of [Ca^2+^]_tip_ in M^2^ of WT (green) and *rbohH-1 rbohJ-2* (red). The variance of the [Ca^2+^]_tip_ is significantly elevated in mutant PTs compared to the WT (*p* = 2.8•10^−10^; Wilcoxon sum rank test). (D) Histogram in arbitrary units of the ratio of the YC3.60 of [Ca^2+^]_tip_/[Ca^2+^]_behind_ in growing PTs of WT (green) and *rbohH-1 rbohJ-2* (red). (E) Histogram of the variance (arbitrary units) of the ratio of [Ca^2+^]_tip_/[Ca^2+^]_behind_ in growing PTs of WT (green) and *rbohH-1 rbohJ-2* (red). The variance of the ratio of [Ca^2+^]_tip_/[Ca^2+^]_behind_ is significantly increased in mutant pollen tubes (*p* = 4.1280•10^−13^, Wilcoxon sum rank test), indicating that the tip-focused Ca^2+^ gradient is less stable in the mutants than in the WT. (F) Histogram of the variance of the growth rates in (mm/s)^2^ for WT (green) and *rbohH-1 rbohJ-2* (red) PTs. Note that the variance of the growth rates is significantly higher in mutant PTs compared to the WT (*p* = 0.008737, Wilcoxon one-sided rank-sum test), indicating that *rbohH-1 rbohJ-2* PT growth is unstable compared to PT growth of the WT.
(TIF)

Figure S11 **External Ca^2+^ partially suppresses pollen tube rupture of** ***rbohH rbohJ*** **mutants.** (A) Quantification of the germination rate of pollen from WT, *rbohH-1 rbohJ-2*, and *rbohH-3 rbohJ-3* plants on different Ca^2+^-containing media. Data are mean ± standard error of the mean (SEM) of three independent experiments with more than 150 pollen grains per genotype and experiment. Single asterisks indicate statistically significant differences from the corresponding control at 5 mM [Ca^2+^] (indicated by #) according to a Student's *t* test with *p*<0.05. (B) Quantification of PT rupture from WT, *rbohH-1 rbohJ-2*, and *rbohH-3 rbohJ-3* plants on different Ca^2+^-containing media. Data are mean ± SEM of three independent experiments with more than 150 pollen grains per genotype and experiment. Single asterisks indicate statistically significant differences from the corresponding control at 5 mM [Ca^2+^] (indicated by #) according to a Student's *t* test with *p*<0.05. (C) Representative images of WT (top) and *rbohH rbohJ* (bottom) pollen grains grown *in vitro* for 5 h on 0 (left) and 15 mM [Ca^2+^] (right). Scale bar = 5 µm.
(TIF)

Table S1 **Parameters of fluorescence recovery after photobleaching (FRAP) at the apical plasma membrane of growing pollen tubes for ANX1-YFP in different backgrounds.**
(DOCX)

Table S2 **Segregation analysis of** ***rboh*** **mutations by PCR-based genotyping or scoring herbicide resistance of the progeny resulting from reciprocal crosses with the wild type (Col-0).**
(DOCX)

Table S3 **Segregation analysis of** ***rboh*** **mutations by PCR-based genotyping in the progeny after self-fertilization.**
(DOCX)

Table S4 **Oligonucleotides used in this study.**
(DOCX)

Text S1 **Supporting protocols.**
(DOCX)

Video S1 **Time-course imaging of plasma membrane invagination in an ANX1-YFP over-expressing pollen tube that ceased to elongate.** Top, bright-field; middle, YFP-derived fluorescence; bottom, FM4-64-derived fluorescence. The focal plane was adjusted between the different frames to focus on the apical membrane growing inwards. Δt = 40 s. Scale bar = 5 µm.
(AVI)

Video S2 **Representative time-course imaging of complemented (top) and ANX1-YFP over-expressing pollen tubes (bottom) treated for 5 min with FM4-64 (2 µM).** Δt = 0.56 s. Scale bar = 5 µm.
(AVI)

Video S3 **Time-course imaging of representative FRAP experiments on two complemented (top) and two ANX1-YFP over-expressing (bottom) pollen tubes.** Bleaching time-stamp occurs at t = 0 s. Green arrow time-stamps appear when 80% of the initial fluorescence is recovered in the apical plasma membrane. Note that ANX1-YFP over-expressing PTs grow slower but recover their fluorescence faster that complemented lines. Δt = 4 s. Scale bar = 5 µm.
(AVI)

Video S4 **Time-course imaging of growing wild-type pollen tube expressing the cytosolic H_2_O_2_-sensitive HyPer.** Top and middle panels show the fluorescence collected between 500 and 540 nm after excitation at 488 nm and 405 nm, respectively. Bottom panel shows the corresponding ratiometric image (F_488_/F_405_). Note the oscillating HyPer activity at the tip of the growing PT. Δt = 3.26 s. Scale bar = 5 µm.
(AVI)

Video S5 **Time-course ratiometric imaging of a young, growing wild-type pollen tube, an arrested wild-type bulge, and a bursting** ***anx1 anx2*** **bulge.** Δt = 4 s. The calibration bar is the same as in Figure 7A. Scale bar = 5 µm. At time = 76 s, a white arrow indicates a sudden increase of [Ca^2+^]_tip_ in *anx1 anx2* before the bulge actually bursts (black arrow at time = 84 s).
(AVI)

Video S6 **Time-course ratiometric imaging of three growing wild-type (left) and three** ***rbohH rbohJ*** **pollen tubes expressing the cytosolic Ca^2+^-sensitive YC3.60.** Δt = 3.26 s. Scale bar = 5 µm.
(AVI)

Video S7 **Time-course imaging of FM4-64 stained wild-type and** ***rbohH rbohJ*** **pollen tubes growing steadily and unsteadily, respectively.** Δt = 1 s. Scale bar = 5 µm.
(AVI)

## Abbreviations

BFA: Brefeldin A
CW: cell wall
ECM: extracellular matrix
FDA: fluorescein diacetate
FRAP: fluorescence recovery after photobleaching
GFP: green fluorescent protein
NADPH: nicotinamide adenine dinucleotide phosphate
PM: plasma membrane
PT: pollen tube
Rboh: respiratory burst oxidase homologue
RLK: receptor-like kinase
ROP, Rho GTPase: of plants
ROS: reactive oxygen species
TE_M_: male transmission efficiency
TE_F_: female transmission efficiency
WT: wild type
YFP: yellow fluorescent protein
