# Supplementary figures and images for: ANXUR Receptor-Like Kinases Coordinate Cell Wall Integrity with Growth at the Pollen Tube Tip Via NADPH Oxidases
Source: PLoS Biol. 2013 Nov 26;11(11):e1001719. doi: 10.1371/journal.pbio.1001719 (PMC3841104; doi:10.1371/journal.pbio.1001719)

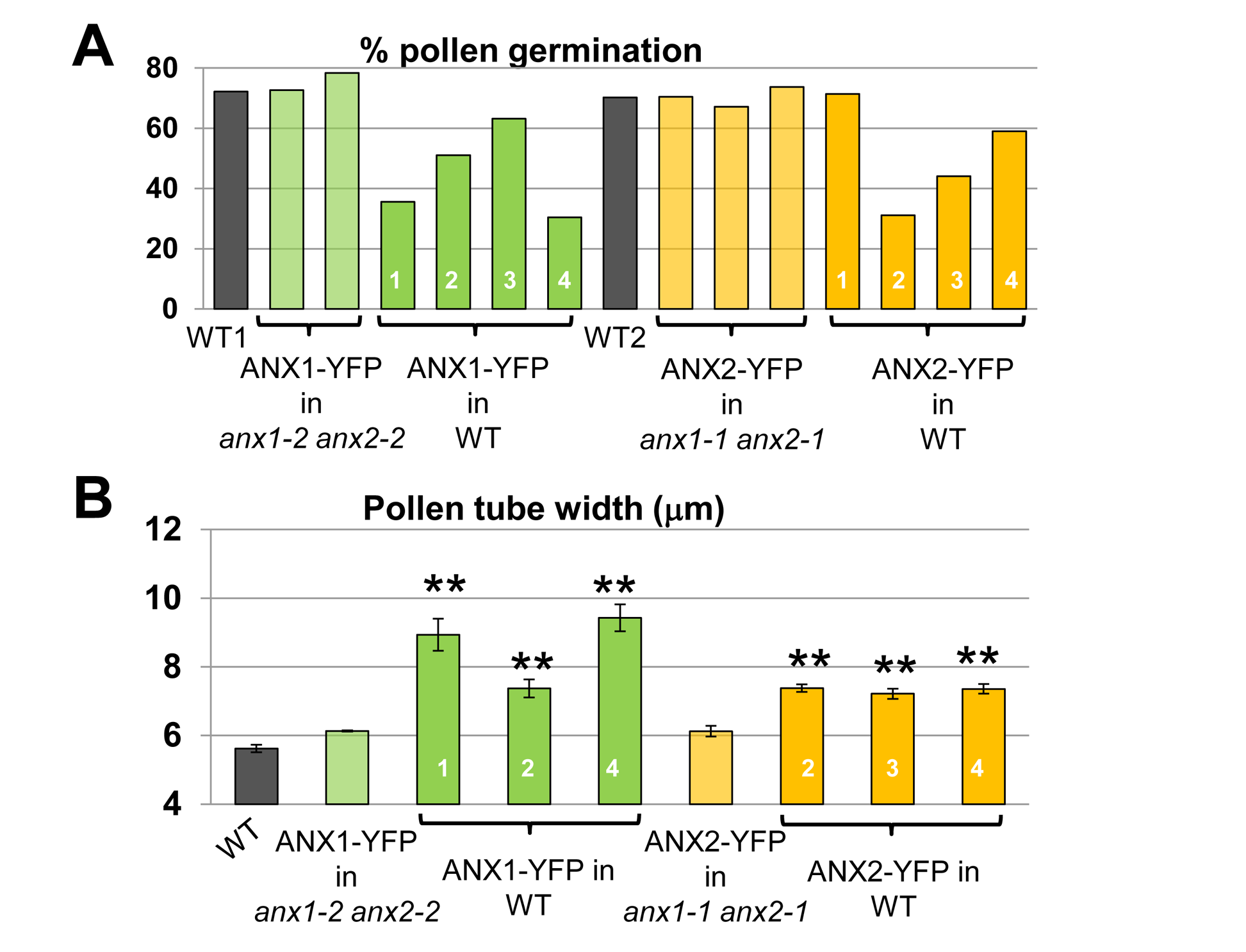

Supplement: Figure S1 — Over-expression of ANX RLKs inhibits pollen germination and increases pollen tube width. (A) Quantification of pollen germination rate after 5 h of in vitro growth for WT, two and three independent ANX1-YFP and ANX2-YFP T3 complemented lines, respectively, as well as four independent T3 ANX1-YFP and ANX2-YFP over-expression lines. Data are representative of three experiments with more than 150 pollen for each genotype. The corresponding PT length measurements are displayed in Figure 1A. (B) Quantification of PT width after 5 h of in vitro growth for WT, one ANX1-YFP, and one ANX2-YFP T3 complemented lines, as well as three independent T3 ANX1-YFP and ANX2-YFP over-expression lines. Data represent mean values ± standard error of the mean (SEM) of three independent experiments with more than 40 PTs per genotype and experiment. Double asterisks indicate statistically significant differences from the WT according to a Student's t test with p<0.01. (TIF) [file pbio.1001719.s001.tif]

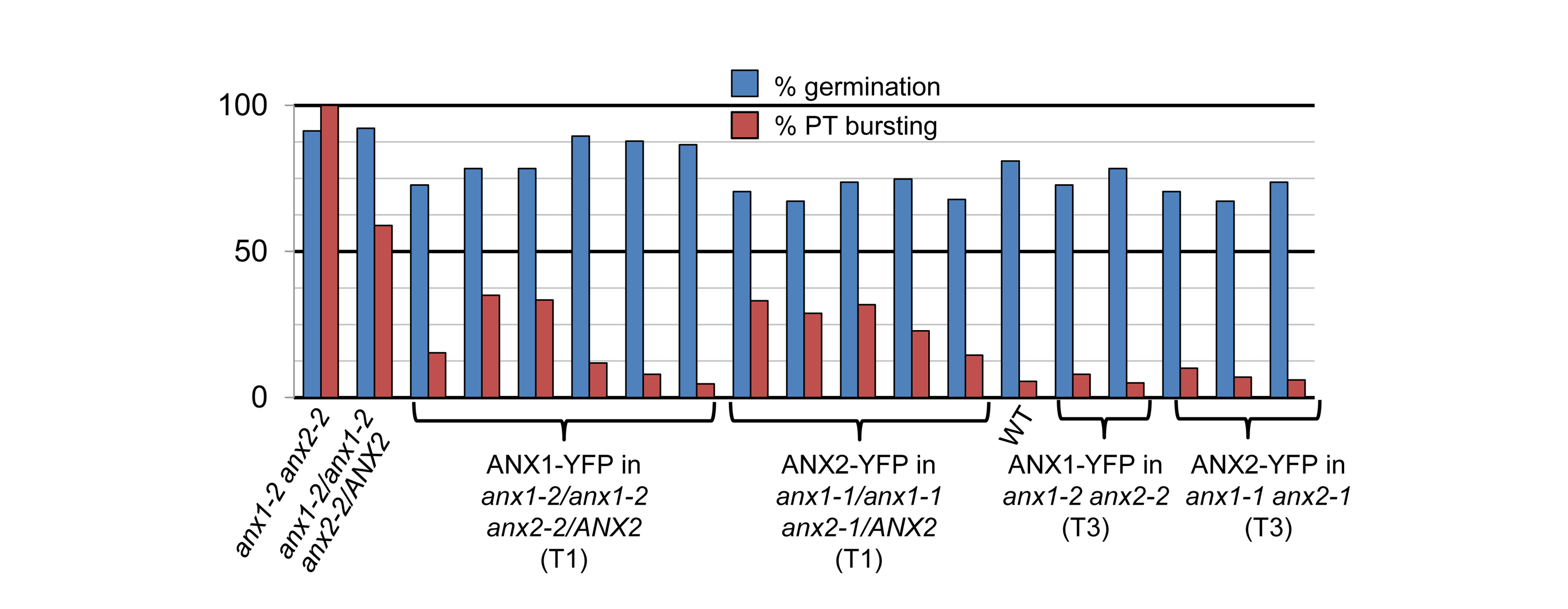

Supplement: Figure S2 — ANX1-YFP and ANX2-YFP fusion proteins complement the anx1 anx2 pollen tube rupture phenotype. Quantification of pollen germination rate and PT rupture after 5 h of in vitro growth for WT, anx1-2 anx2-2, anx1-2/anx1-2 anx2-2/ANX2, 6, and 5 independent T1 lines of ANX1-YFP in anx1-2/anx1-2 anx2-2/ANX2 and ANX2-YFP in anx1-1/anx1-1 anx2-1/ANX2, respectively, as well as two and three independent T3 homozygous ANX1-YFP in anx1-2 anx2-2 and ANX2-YFP in anx1-1 anx2-1 complemented lines, respectively. More than 150 pollen were analyzed per genotype. (TIF) [file pbio.1001719.s002.tif]

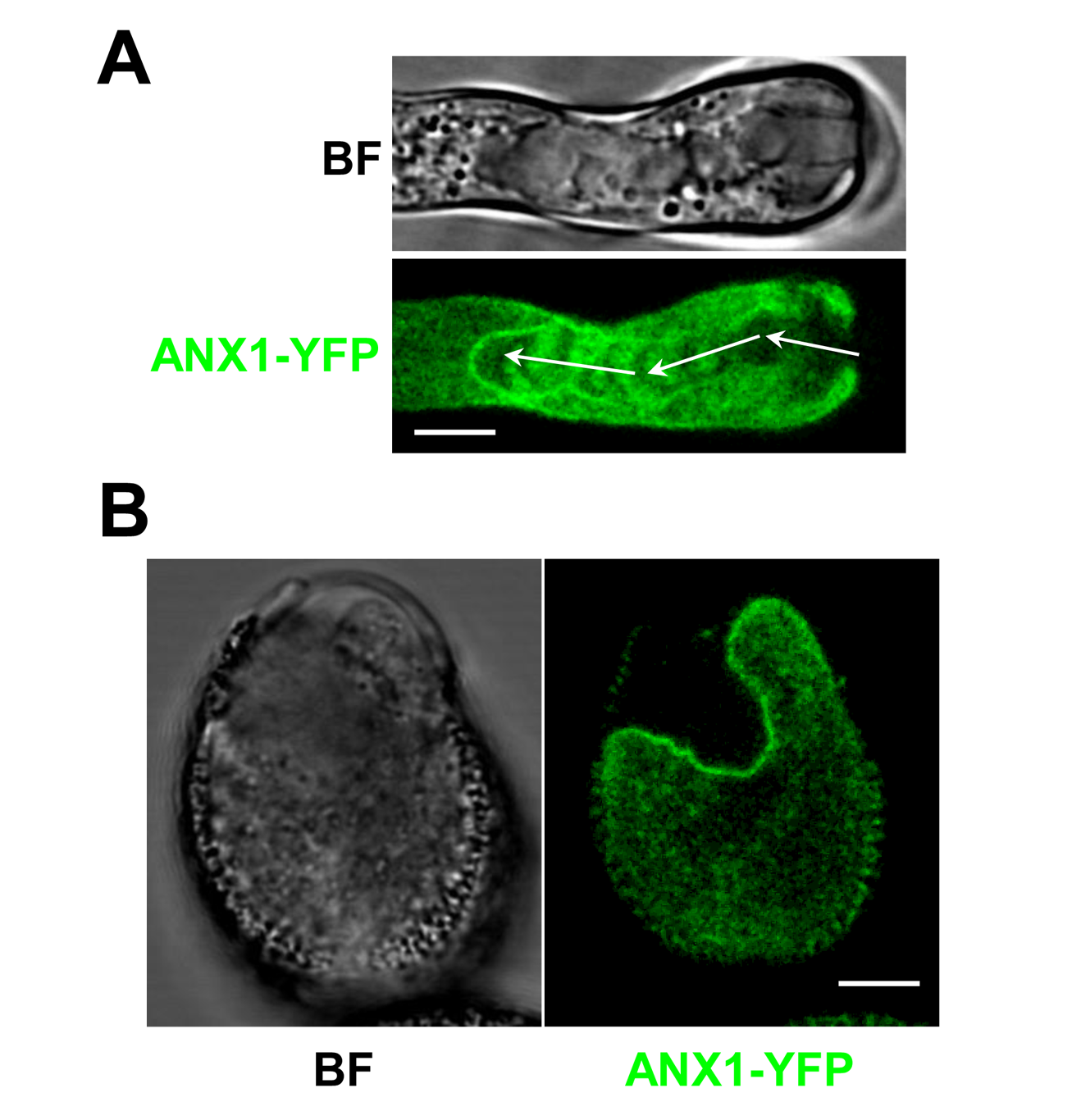

Supplement: Figure S3 — ANX RLK over-expression triggers plasma membrane invagination. (A) Median plane confocal image of an ANX1-YFP over-expressing PT, in which the apical membrane grows inwards. Filters are indicated on the left. Scale bar = 5 µm. (B) Single plane confocal image of plasma membrane invagination in an ANX1-YFP over-expressing pollen grain. (TIF) [file pbio.1001719.s003.tif]

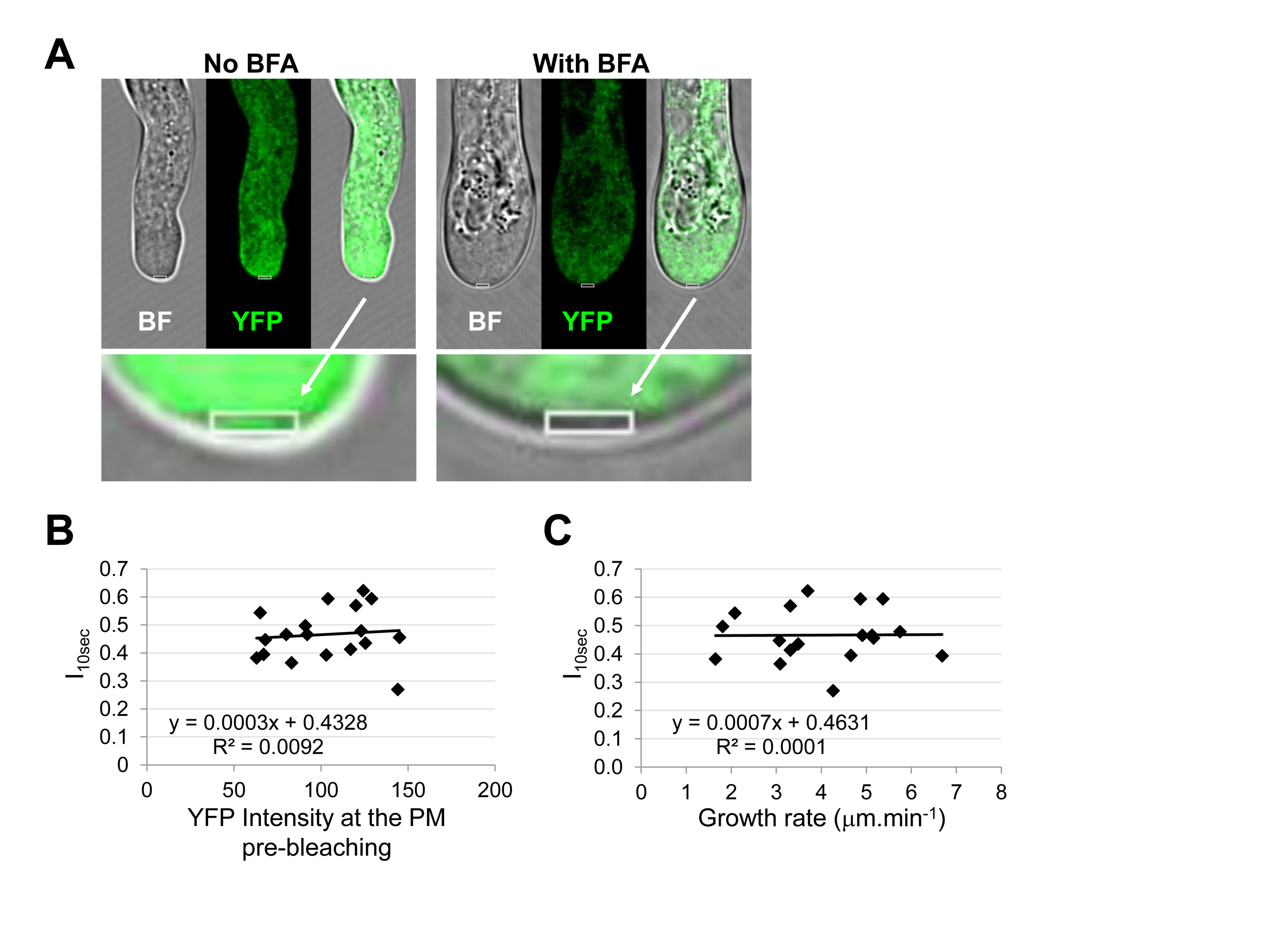

Supplement: Figure S4 — BFA treatment disrupts the enrichment of ANX1-YFP at the apical plasma membrane. (A) Representative median plane sections of anx1 anx2 complemented PTs expressing ANX1-YFP with (right) or without (left) BFA treatment. Note that the YFP-derived fluorescence is much weaker in the apical membrane-derived region of interest of the BFA-treated PTs compared to that of non-treated PTs (left). The same regions of interest (ROIs) were used for FRAP experiments. No correlation between relative fluorescence recovery 10 s after photobleaching and original amount of fluorescent protein in the apical plasma membrane (B) nor with PT growth rate (C). (TIF) [file pbio.1001719.s004.tif]

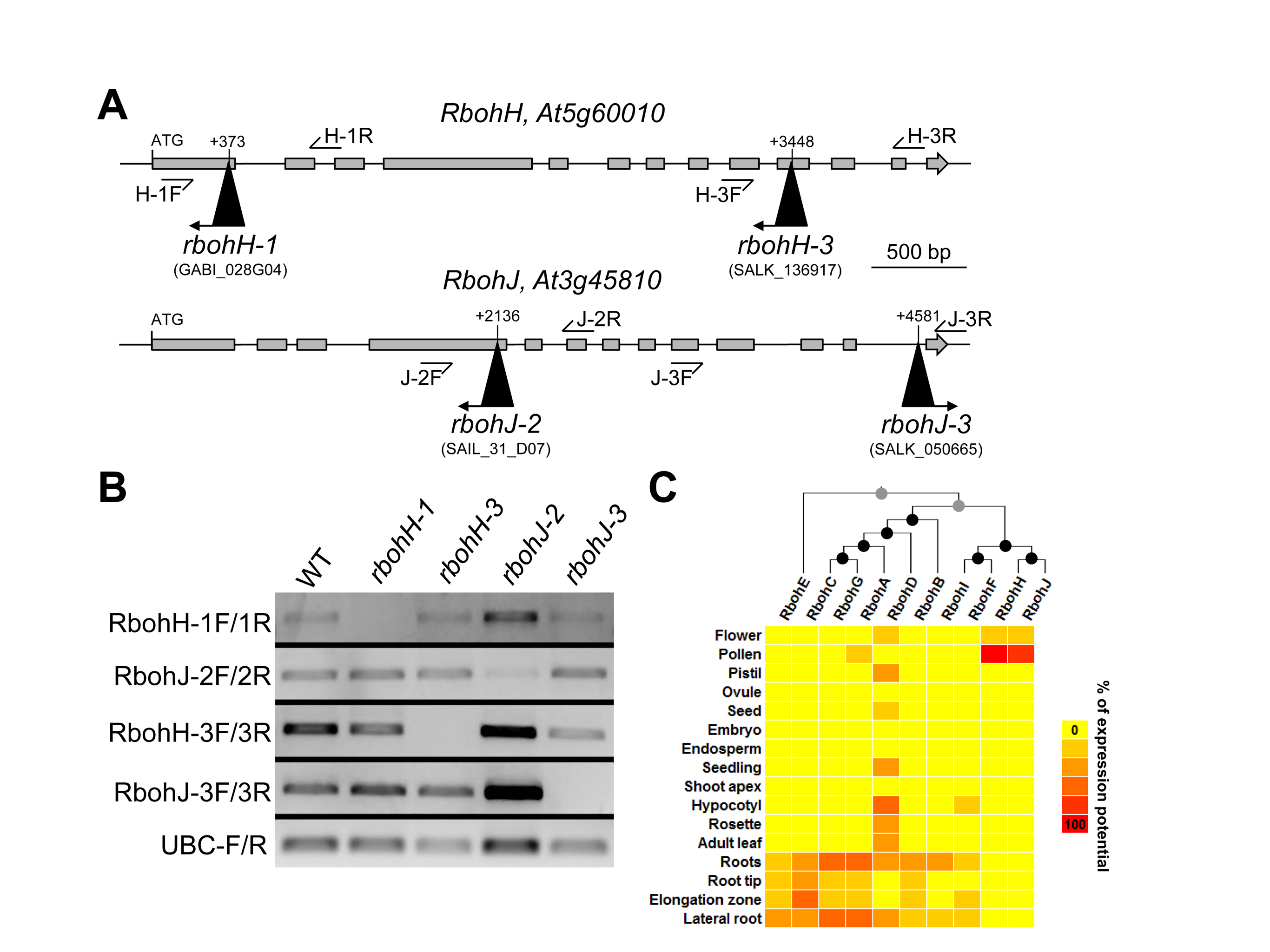

Supplement: Figure S5 — Structure and expression of Rboh genes in Arabidopsis . (A) The genomic organization of the pollen-expressed NADPH oxidase genes RbohH and RbohJ and positions of the rbohH-1, rbohH-3, rbohJ-2, and rbohJ-3 T-DNA insertions. The orientation of the left border sequence of the respective T-DNAs is represented by black arrows. The positions of the primers used to genotype the mutants are indicated. (B) RT-PCR analyses of cDNAs from open-flowers show no RbohH transcripts in the T-DNA insertion lines rbohH-1 and rbohH-3. There are much less or no RbohJ transcripts in the T-DNA insertion lines rbohJ-2 and rbohJ-3, respectively. UBC21 (At5g25760) was used as a control. Amplification was performed for 30 cycles for UBC21 and for 35 cycles for RbohH and RbohJ. (C) Multiple alignments of Arabidopsis Rboh proteins were performed with ClustalW 1.83 and the phylogenetic tree was reconstructed with MEGA4 using the protein sequence parsimony method (bootstrap test, 1,000 replicates). Black and grey circles at nodes indicate bootstrap values of more than 900 and between 800 and 900, respectively. The HsNOX5 was used as outgroup. The tree was then combined with the relative gene expression of Arabidopsis Rboh family members in various plant tissues according to the Genevestigator microarray database using the Meta-Profile Analysis tool, Anatomy Profile [59]. (TIF) [file pbio.1001719.s005.tif]

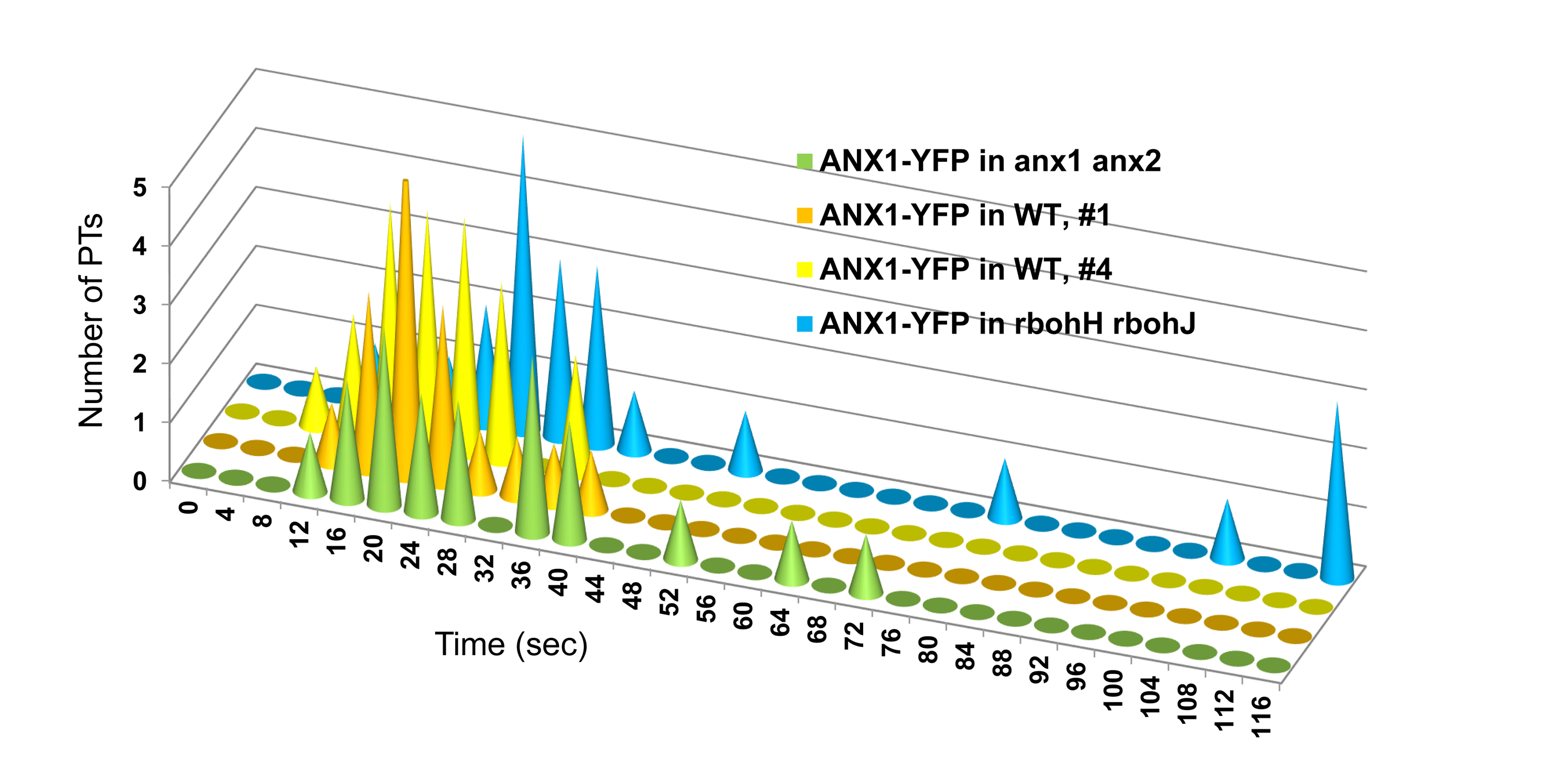

Supplement: Figure S6 — Distribution of pollen tubes of ANX1-YFP in anx1 anx2 , in wild-type (over-expressor line #1 and #4), and rbohH rbohJ backgrounds relative to the time required to recover 80% of the initial fluorescence. Unlike PTs from complemented and over-expressor lines, some rbohH rbohJ PTs expressing ANX1-YFP were not able to recover 80% of the initial fluorescence at the apical plasma membrane. (TIF) [file pbio.1001719.s006.tif]

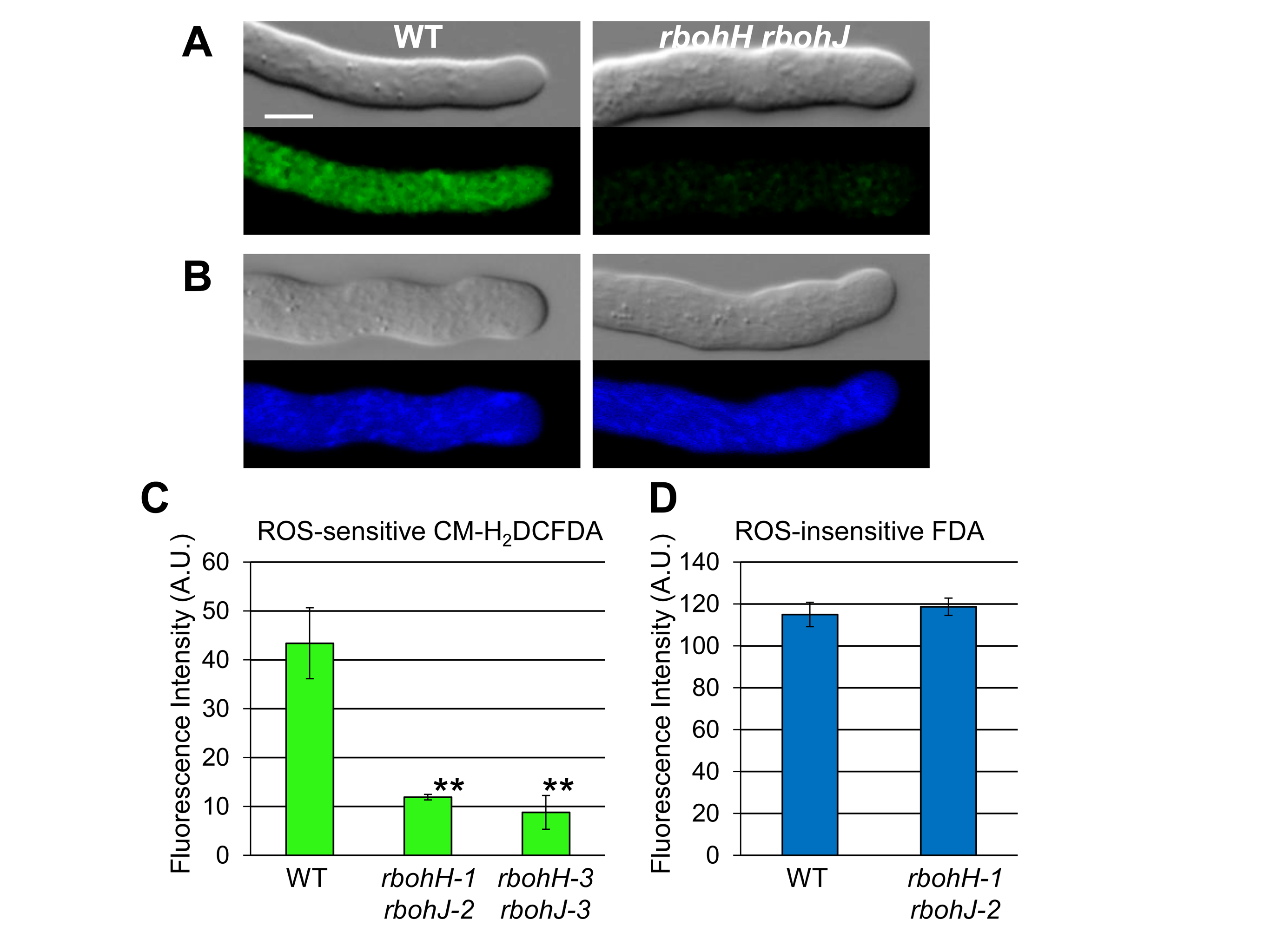

Supplement: Figure S7 — rbohH rbohJ pollen tubes display decreased levels of ROS-sensitive CM-H2DCFDA-derived fluorescence compared to the wild type. (A) Single median plane images of growing WT and rbohH rbohJ PTs stained with the ROS-sensitive CM-H2DCFDA dye and imaged with the same settings. Scale bar = 5 µm. (B) Single median plane images of growing WT and rbohH rbohJ PTs stained with the ROS-insensitive FDA dye and imaged with the same settings. The scale is the same as in (A). (C) Quantification of ROS-sensitive CM-H2DCFDA-derived fluorescence in a circle with 4 µm diameter at the tip of growing WT and rbohH rbohJ PTs. Data are mean ± standard error of the mean (SEM) of three independent experiments with more than eight PTs per genotype and experiment. Double asterisks indicate significant differences from the WT according to a Student's t test with p<0.01. (D) Quantification of ROS-insensitive FDA-derived fluorescence in a circle with 4 µm diameter at the tip of growing WT and rbohH rbohJ PTs. Data are mean ± SEM of three independent experiments with more than eight PTs per genotype and experiment. (TIF) [file pbio.1001719.s007.tif]

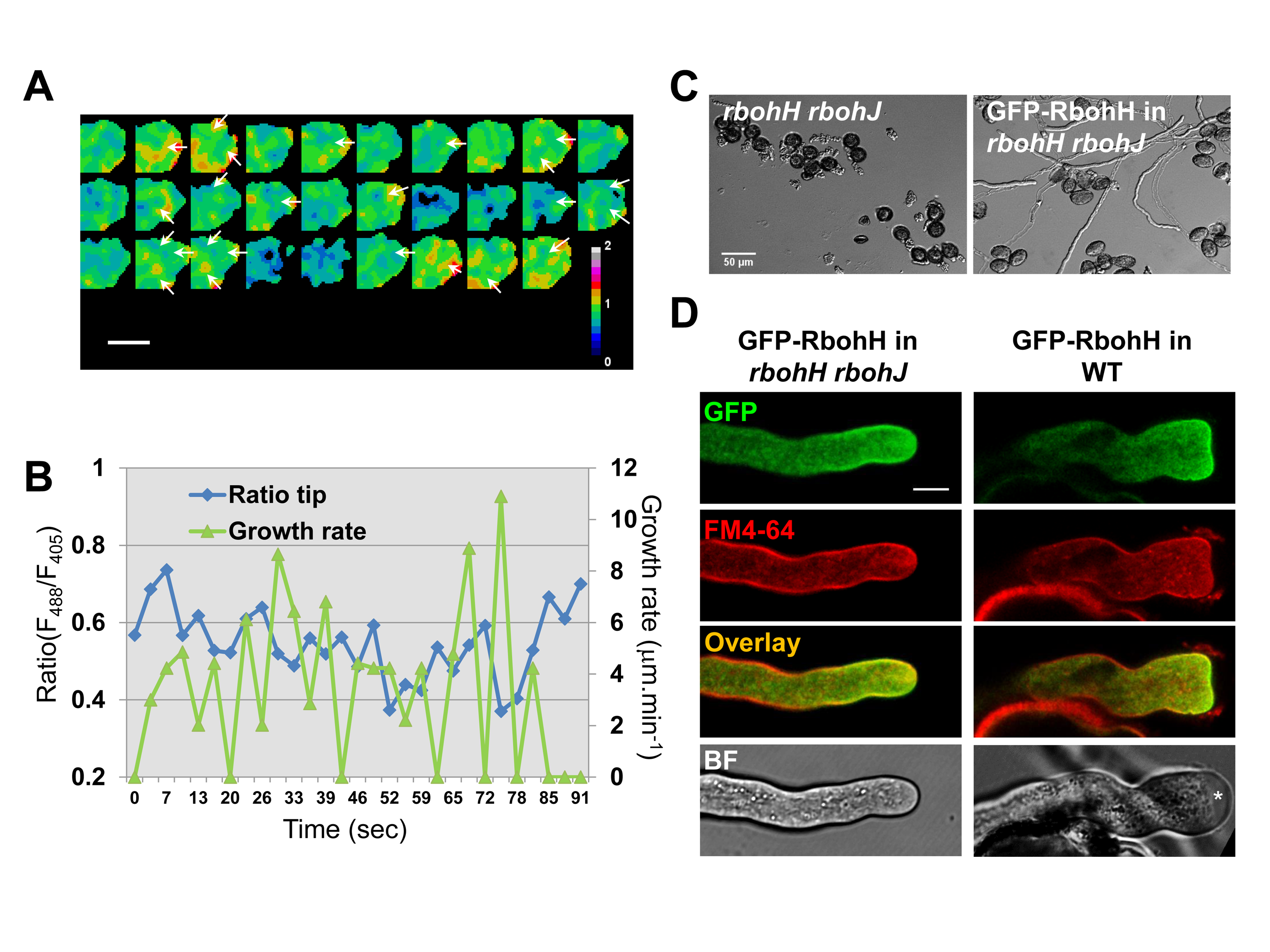

Supplement: Figure S8 — Pulsating H2O2-sensitive HyPer activity at the tip of growing wild-type pollen tubes correlates with GFP-RbohH localization. (A) Time-course ratiometric imaging of the tip of growing WT PT expressing cytosolic HyPer and the corresponding histogram (B) displaying the ratio (F488/F405) at the tip (blue line) over 90 s, as well as the PT growth rates (green line). Scale bar = 5 µm. (C) In vitro PT growth assay showing that the GFP-RbohH protein fusion complements the rbohH rbohJ PT bursting phenotype in T1 rbohH-3 rbohJ-3 heterozygous for GFP-RbohH. Left, rbohH-3 rbohJ-3 pollen. Right, pollen of a representative T1 rbohH-3 rbohJ-3 line expressing GFP-RbohH. (D) Representative single median plane images of a normally growing PT of a GFP-RbohH complemented line (left) and an arrested PT of an GFP-RbohH over-expressing line with apical membrane invagination (right) and over-accumulation of CW material (asterisk). The different filters are indicated. Before imaging, PTs were treated for 5 min with germination liquid medium containing FM4-64 (2 µM). Scale bar = 5 µm. (TIF) [file pbio.1001719.s008.tif]

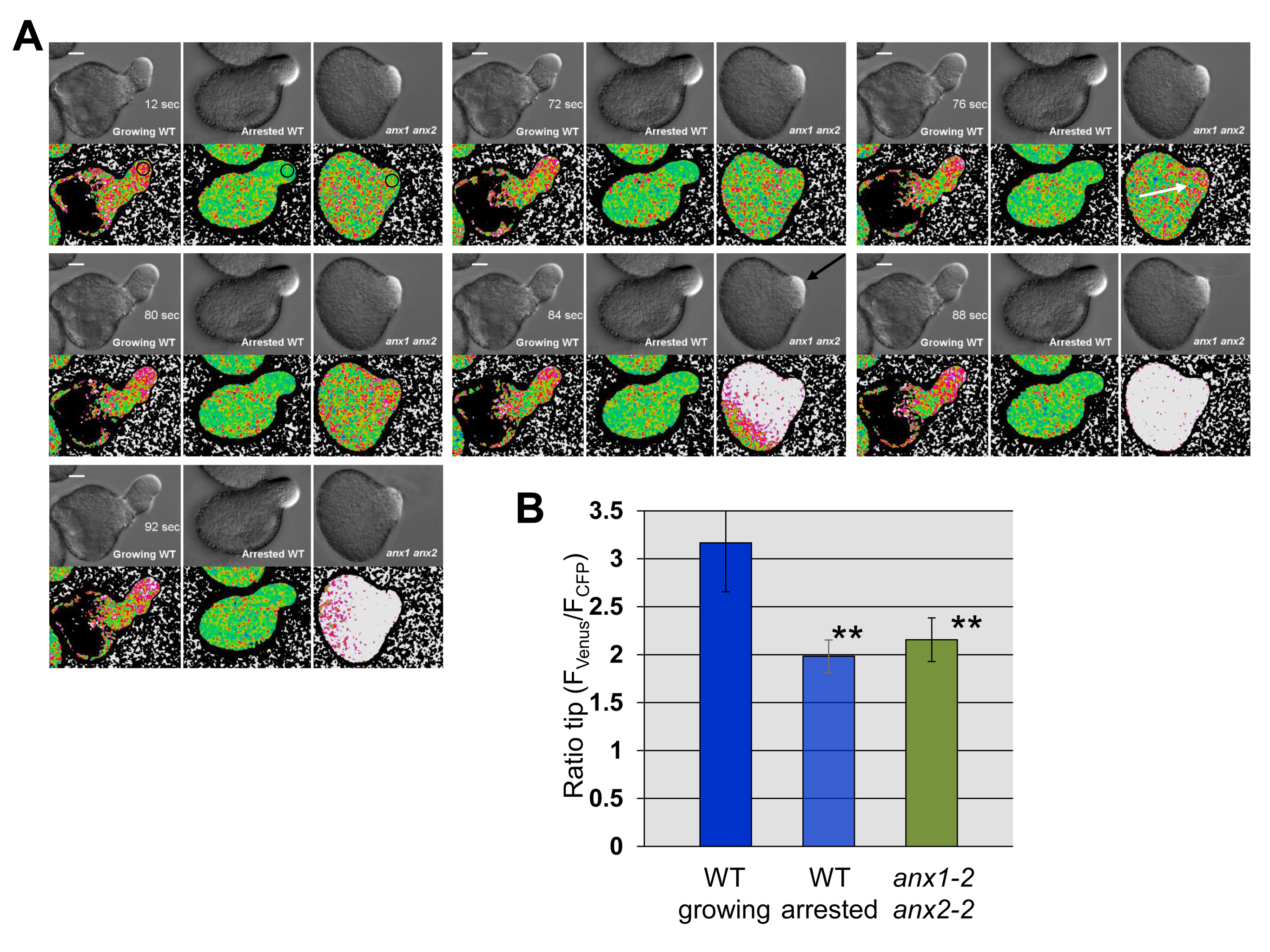

Supplement: Figure S9 — Ca2+− sensitive cameleon YC3.60 ratiometric imaging shows that [Ca2+]cyt levels are decreased in anx1 anx2 bulges compared to young, growing wild-type pollen tubes but are similar to arrested wild-type bulges. (A) Representative ratiometric images of young growing WT PT, arrested WT bulge, and bursting anx1 anx2 bulge expressing cytosolic YC3.60. On the ratiometric images, the black circles represent the region of interests of 4 µm diameter used for measurements at the PT tip. At time = 76 s, a white arrow indicates a sudden increase of [Ca2+]tip in anx1 anx2 before the bulge actually bursts (black arrow at time = 84 s). Note how external Ca2+ enters the anx1 anx2 bulge once it has ruptured. See also corresponding Video S5. The calibration bar is the same as in Figure 7A. Scale bar = 5 µm. (B) Quantification of YC3.60 ratio (FCFP/FVenus) at the tip of young, growing WT PTs, arrested WT bulges, and anx1 anx2 bulges before rupture (n>15 for each category). Data are shown as the mean of ratios over 90 s±standard deviation (SD). Double asterisks indicate significant differences from the growing WT PTs according to a Student's t test with p<0.01. (TIF) [file pbio.1001719.s009.tif]

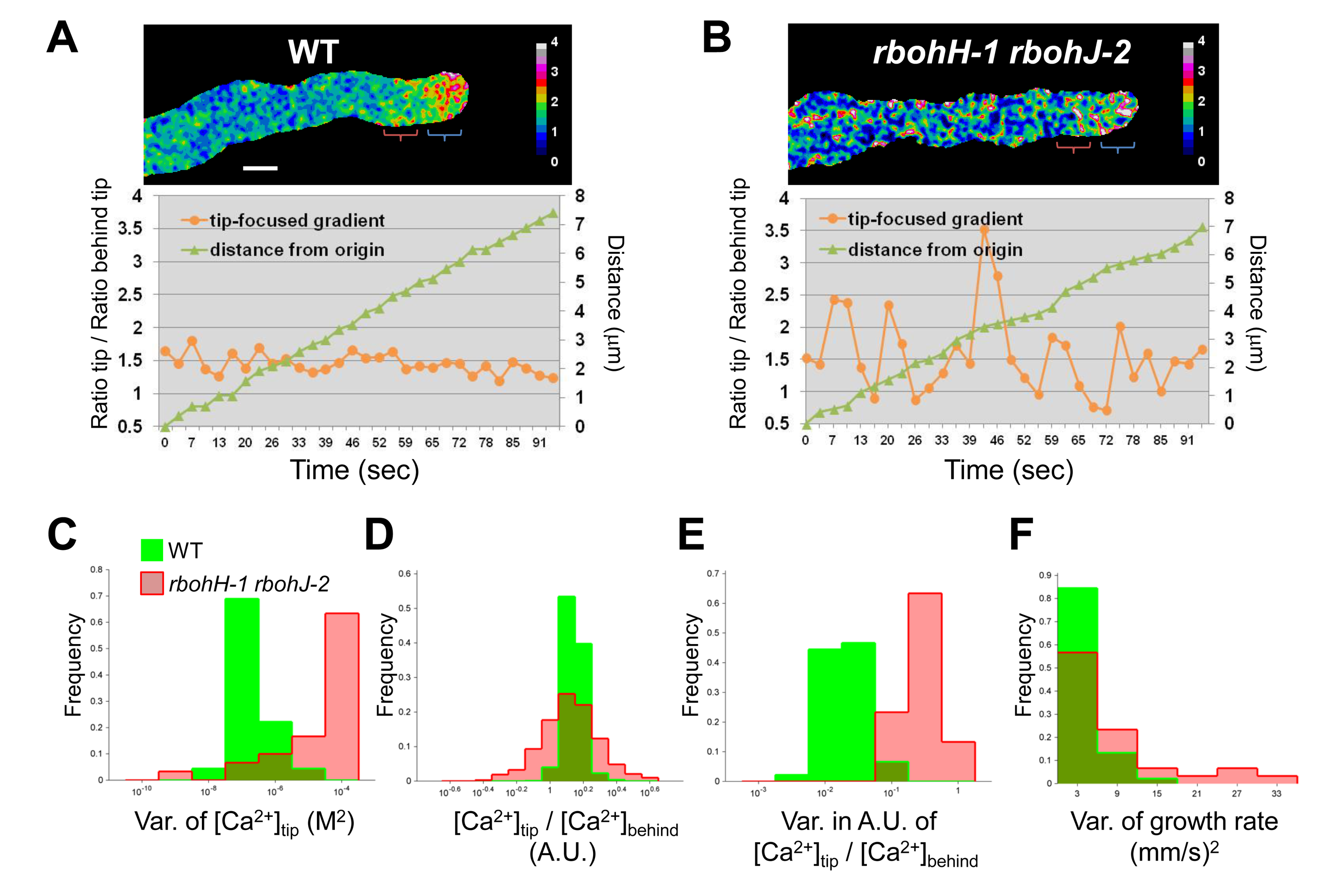

Supplement: Figure S10 — Tip-focused Ca2+ gradient and pollen tube growth rates are less stable in rbohH-1 rbohJ-2 than in the wild type. Representative images of growing WT (A) and rbohH-1 rbohJ-2 (B) PTs expressing cytosolic YC3.60 and the corresponding histograms displaying the ratio of [Ca2+]tip/[Ca2+]behind (i.e., tip-focused Ca2+ gradient, orange line) over 90 s, and the travelled distance of the PT tip (green line). Note how both the tip-focused Ca2+ gradient and the PT growth rate are more stable in WT compared to the mutant. Scale bar = 5 µm. (C) Histogram of the variance of [Ca2+]tip in M2 of WT (green) and rbohH-1 rbohJ-2 (red). The variance of the [Ca2+]tip is significantly elevated in mutant PTs compared to the WT (p = 2.8•10−10; Wilcoxon sum rank test). (D) Histogram in arbitrary units of the ratio of the YC3.60 of [Ca2+]tip/[Ca2+]behind in growing PTs of WT (green) and rbohH-1 rbohJ-2 (red). (E) Histogram of the variance (arbitrary units) of the ratio of [Ca2+]tip/[Ca2+]behind in growing PTs of WT (green) and rbohH-1 rbohJ-2 (red). The variance of the ratio of [Ca2+]tip/[Ca2+]behind is significantly increased in mutant pollen tubes (p = 4.1280•10−13, Wilcoxon sum rank test), indicating that the tip-focused Ca2+ gradient is less stable in the mutants than in the WT. (F) Histogram of the variance of the growth rates in (mm/s)2 for WT (green) and rbohH-1 rbohJ-2 (red) PTs. Note that the variance of the growth rates is significantly higher in mutant PTs compared to the WT (p = 0.008737, Wilcoxon one-sided rank-sum test), indicating that rbohH-1 rbohJ-2 PT growth is unstable compared to PT growth of the WT. (TIF) [file pbio.1001719.s010.tif]

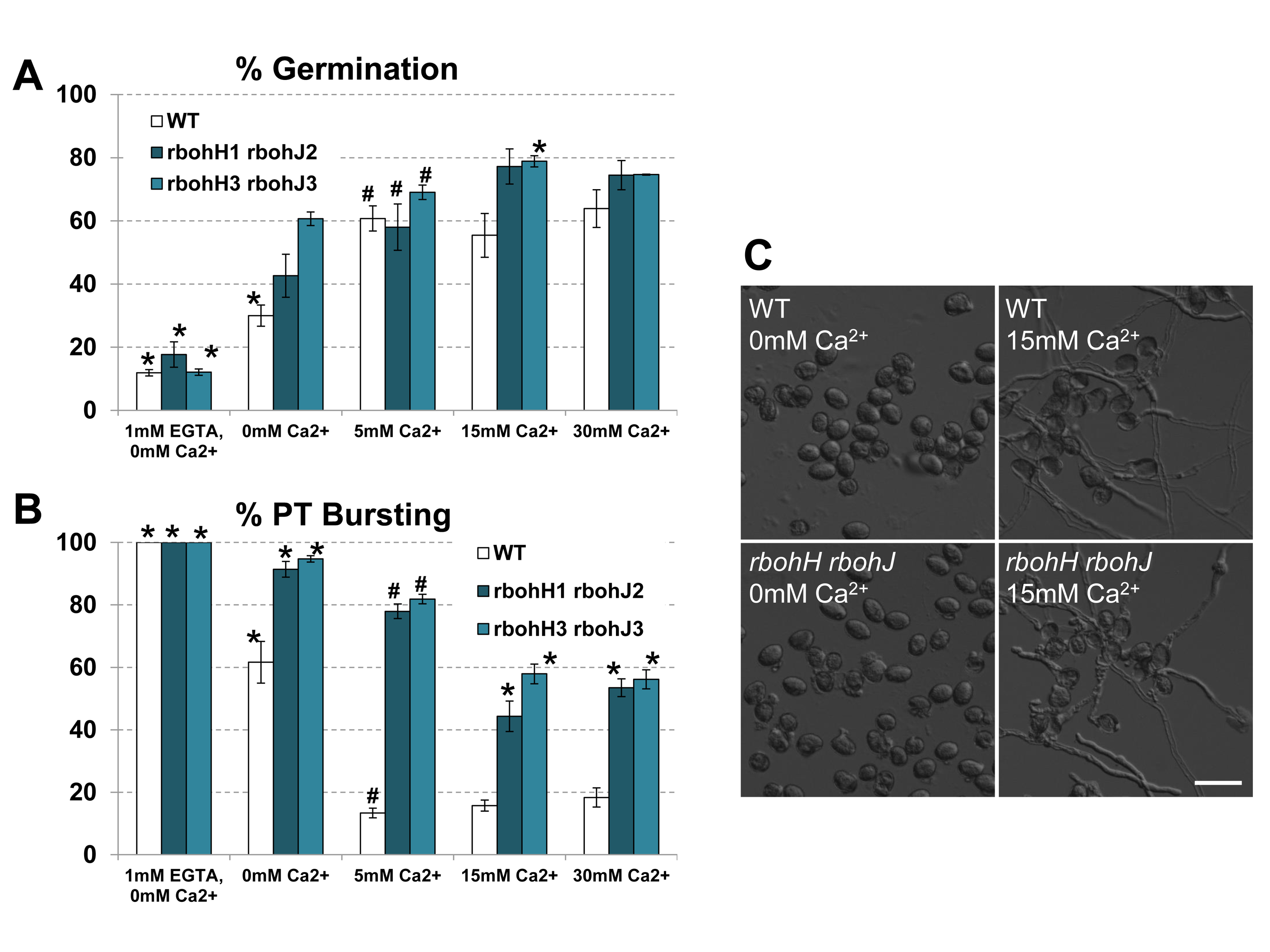

Supplement: Figure S11 — External Ca2+ partially suppresses pollen tube rupture of rbohH rbohJ mutants. (A) Quantification of the germination rate of pollen from WT, rbohH-1 rbohJ-2, and rbohH-3 rbohJ-3 plants on different Ca2+-containing media. Data are mean ± standard error of the mean (SEM) of three independent experiments with more than 150 pollen grains per genotype and experiment. Single asterisks indicate statistically significant differences from the corresponding control at 5 mM [Ca2+] (indicated by #) according to a Student's t test with p<0.05. (B) Quantification of PT rupture from WT, rbohH-1 rbohJ-2, and rbohH-3 rbohJ-3 plants on different Ca2+-containing media. Data are mean ± SEM of three independent experiments with more than 150 pollen grains per genotype and experiment. Single asterisks indicate statistically significant differences from the corresponding control at 5 mM [Ca2+] (indicated by #) according to a Student's t test with p<0.05. (C) Representative images of WT (top) and rbohH rbohJ (bottom) pollen grains grown in vitro for 5 h on 0 (left) and 15 mM [Ca2+] (right). Scale bar = 5 µm. (TIF) [file pbio.1001719.s011.tif]
