# Supplementary material for: ANXUR Receptor-Like Kinases Coordinate Cell Wall Integrity with Growth at the Pollen Tube Tip Via NADPH Oxidases
Source: PLoS Biol. 2013 Nov 26;11(11):e1001719. doi: 10.1371/journal.pbio.1001719 (PMC3841104; doi:10.1371/journal.pbio.1001719)
Supplement: Table S1 — Parameters of fluorescence recovery after photobleaching (FRAP) at the apical plasma membrane of growing pollen tubes for ANX1-YFP in different backgrounds. (DOCX) [file pbio.1001719.s012.docx]

**Table S1. Parameters of fluorescence recovery after photobleaching (FRAP) at the apical plasma membrane of growing pollen tubes for ANX1-YFP in different backgrounds.**

| Genotype | R^2^ (curve fit) | I_0_ | I_Inf_ | τ_1/2_ | I_10sec_ | Growth rate (μm min^-1^) | PTs |
| --- | --- | --- | --- | --- | --- | --- | --- |
| ANX1-YFP in a*nx1 anx2* | 0.9 ± 0.05 | 0.01 ± 0.03 | 0.966 ± 0.15 | 11.27 ± 3.42 | 0.47 ± 0.09 | 4.02 ± 1.41 | 18 |
| ANX1-YFP in WT, #1 | 0.96 ± 0.03 ** | 0.1 ± 0.07 | 1 ± 0.1 | 9.78 ± 3.68 | 0.57 ± 0.1 ** | 1.34 ± 0.68 ** | 17 |
| ANX1-YFP in WT, #4 | 0.94 ± 0.05 * | 0.09 ± 0.1 | 0.97 ± 0.09 | 9.3 ± 3.6 | 0.57 ± 0.13 * | 1.78 ± 0.53 ** | 20 |
| ANX1-YFP in *rbohH rbohJ* | 0.73 ± 0.13 ** | 0.01 ± 0.03 | 0.87 ± 0.18 | 10.43± 5.11 | 0.45 ± 0.09 | 2.63 ± 1.53 ** | 23 |

Experimental data for relative fluorescence intensity recovery at time t was fitted with the exponential equation:

I(t)=I_0_+(I_inf_ –I_0_)*(1-2^(-t/ τ1/2)^)

where I_0_ is the relative fluorescence right after photobleaching, I_inf_ is the maximum relative recoverable intensity and τ_1/2_ is the time when half of the recoverable fluorescence is recovered. Because τ_1/2_ for all genotypes was around 10 sec, we calculated and compared the relative intensity recovery 10 sec after photobleaching.

** and * denotes statistical significance at P<0.01and P<0.05, respectively, compared to the complemented line, using a two-tailed equal variance Student t-Test. Averages are shown ± standard error.
