# Supplementary material for: ANXUR Receptor-Like Kinases Coordinate Cell Wall Integrity with Growth at the Pollen Tube Tip Via NADPH Oxidases
Source: PLoS Biol. 2013 Nov 26;11(11):e1001719. doi: 10.1371/journal.pbio.1001719 (PMC3841104; doi:10.1371/journal.pbio.1001719)
Supplement: Table S2 — Segregation analysis of rboh mutations by PCR-based genotyping or scoring herbicide resistance of the progeny resulting from reciprocal crosses with the wild type (Col-0). (DOCX) [file pbio.1001719.s013.docx]

**Table S2. Segregation analysis of *rboh* mutations by PCR-based genotyping or scoring herbicide resistance of the progeny resulting from reciprocal crosses with the wild type (Col-0).**

| Female  x  Male | ***Rboh/Rboh***  *or*  *rbohH/****RbohH*** ***RbohJ/RbohJ***  (a) | *rboh/****Rboh***  *or*  *rbohH/****RbohH*** *rbohJ****/RbohJ***  (b) | TE (%) |
| --- | --- | --- | --- |
| *rbohH-1/****RbohH***  x  Col-0 | 109 | 127 | **116.5** |
| Col-0  x  *rbohH-1/****RbohH*** | 126 | 102 | **81** |
| *rbohJ-2/****RbohJ***  x  Col-0 | 116 | 126 | **108.6** |
| Col-0  x  *rbohJ-2/****RbohJ*** | 125 | 115 | **92** |
| *rbohH-1/rbohH-1 rbohJ-2/****RbohJ***  x  Col-0 | 123 | 114 | **92.7** |
| Col-0  x  *rbohH-1/rbohH-1 rbohJ-2/****RbohJ*** | 209 | 9 | **4.3*** |
| *rbohH-1/****RbohH*** *rbohJ-2/rbohJ-2*  x  Col-0 | 121 | 110 | **90.9** |
| Col-0  x  *rbohH-1/****RbohH*** *rbohJ-2/rbohJ-2* | 212 | 16 | **7.5*** |
| *rbohH-3/rbohH-3 rbohJ-3/****RbohJ***  x  Col-0 | 52 | 45 | **86.5** |
| Col-0  x  *rbohH-3/rbohH-3 rbohJ-3/****RbohJ*** | 186 | 2 | **1.1*** |

Transmission efficiency: TE=(b/a) x 100 %; if the mutant genotype was used as the female this corresponds to TE_F_, if it was used as a male to TE_M_.

*denotes statistically significant difference from the expected 1:1 ratio for normal transmission with P<0.0001 (Two-tailed exact Fisher’s test).

For clarity, WT alleles are shown in bold.
