# Supplementary material for: ANXUR Receptor-Like Kinases Coordinate Cell Wall Integrity with Growth at the Pollen Tube Tip Via NADPH Oxidases
Source: PLoS Biol. 2013 Nov 26;11(11):e1001719. doi: 10.1371/journal.pbio.1001719 (PMC3841104; doi:10.1371/journal.pbio.1001719)
Supplement: Table S3 — Segregation analysis of rboh mutations by PCR-based genotyping in the progeny after self-fertilization. (DOCX) [file pbio.1001719.s014.docx]

**Table S3. Segregation analysis of *rboh* mutations by PCR-based genotyping in the progeny after self-fertilization.**

| Genotype | ***RbohH****/****RbohH*** *rbohJ/rbohJ* | | *rbohH/****RbohH*** *rbohJ/rbohJ* | *rbohH/rbohH rbohJ/rbohJ* | Ratios |
| --- | --- | --- | --- | --- | --- |
| *rbohH-1/****RbohH*** *rbohJ-2/rbohJ-2* | | 90 | 80 | 7 | **1:0.88:0.07*** |
| *rbohH-3/****RbohH*** *rbohJ-3/rbohJ-3* | | 103 | 82 | 3 | **1:0.80:0.03*** |

*denotes statistically significant difference from the expected 1:2:1 ratio with P<0.0001 (two-tailed χ^2^ test). For clarity, WT alleles are shown in bold.
