# Supplementary material for: ANXUR Receptor-Like Kinases Coordinate Cell Wall Integrity with Growth at the Pollen Tube Tip Via NADPH Oxidases
Source: PLoS Biol. 2013 Nov 26;11(11):e1001719. doi: 10.1371/journal.pbio.1001719 (PMC3841104; doi:10.1371/journal.pbio.1001719)
Supplement: Table S4 — Oligonucleotides used in this study. (DOCX) [file pbio.1001719.s015.docx]

**Table S4. Oligonucleotides used in this study.**
