# Supplementary material for: ANXUR Receptor-Like Kinases Coordinate Cell Wall Integrity with Growth at the Pollen Tube Tip Via NADPH Oxidases
Source: PLoS Biol. 2013 Nov 26;11(11):e1001719. doi: 10.1371/journal.pbio.1001719 (PMC3841104; doi:10.1371/journal.pbio.1001719)
Supplement: Text S1 — Supporting protocols. (DOCX) [file pbio.1001719.s016.docx]

**Supporting Protocols**

## Plant material, growth conditions and mutant genotyping

Plant growth conditions, transformation and RT-PCR analyses were rigorously as reported previously (Boisson-Dernier, et al., 2009). All plants used in this study are from the Columbia ecotype. All primers used in this study are listed in Table S4. The pACA9-ANX1-YFP and pACA9-ANX2-YFP constructs in the binary ps779 vector conferring Hygromycin resistance were described previously (Boisson-Dernier, et al., 2009). T-DNA insertion lines *rbohH-1* and *rbohH-3* for the *RbohH* gene (*At5g60010*) as well as *rbohJ-2* and *rbohJ-3* for the *RbohJ* gene (*At3g45810*) were obtained from ABRC and correspond to the GABI_028G04 (373 bp downstream of ATG, Sulfadiazine resistance), SALK_136917 (3448 bp downstream of ATG), SAIL_31_D07 (2136 bp downstream of ATG, Basta resistance) and SALK_050665 (4581 downstream of ATG), lines respectively. Genotyping PCR reactions for *rbohH-1* and *rbohH-3* were performed with primer pairs RbohH-F1/RbohH-R1, RbohH-F1/LBgabi2, RbohH-F5/RbohH-R5 and RbohH-F5/LBa1. For *rbohJ-2* and *rbohJ-3* genotyping, primer pairs RbohJ-F2/RbohJ-R2, RbohJ-F2/LBsail1, RbohJ-F3/RbohJ-R3 and LBa1/ RbohJ-R3 were used. WT plants expressing pACT1-YC3.60 (Iwano, et al., 2009) were crossed as male to *rbohH-1 rbohJ-2* plants, and partially male sterile *rbohH-1 rbohJ-2* plants homozygous for pACT1-YC3.60 were selected in further F3 generation.

## In vitro pollen growth assays and aniline blue staining

*In vitro* pollen growth assays were carried out as described previously (Boavida and McCormick, 2007). Briefly, one day opened flowers were incubated at 22ºC for 30 minutes in moisture incubation boxes, then brushed on the germination medium (0.01% boric acid, 5 mm CaCl_2_, 5 mm KCl, 1 mm MgSO_4_, 10% sucrose pH 7.5, 1.5% low-melting agarose)-containing slides. Slides in moisture incubation boxes were pre-incubated for 45 minutes at 30ºC before returning them at 22ºC for several hours. Before imaging, a drop of liquid germination medium was applied to the slides and a cover slip was gently applied. To determine the percentage of ruptured pollen tubes (PTs), PT length and width measurements, pollen grains and PTs were imaged with a Leica DM6000 and analyzed using the ImageJ 1.47d software (<http://rsb.info.nih.gov/ij>). Aniline blue staining was performed as previously described (Huck, et al., 2003).

## HyPer cloning

The HyPer cDNA was amplified by PCR with primers HyPer-*Spe*I and HyPer-*Sac*I using the Phusion® DNA polymerase (Finnzymes) from the purchased pHyPer–Cyto vector (Evrogen). The amplicons were cloned into pGEM^®^-T Easy vector (Promega) and sequenced, then digested with *Spe*I and *Sac*I, and inserted instead of GFP into binary pACA9-GFP ps536 that confers resistance to Basta (Schiøtt, et al., 2004). Around forty T1 Basta-resistant plants were recovered, which displayed various levels of HyPer-derived fluorescence in pollen. One T1 line was selected for displaying a high HyPer expression level in pollen and ~75% Basta resistance in its progeny, and propagated further to generate homozygous pACA9-HyPer expressing wild-type (WT) plants. pACA9-HyPer expressing WT plants were crossed as male to *rbohH-1 rbohJ-2* double mutant plants, and partially male sterile *rbohH-1 rbohJ-2* plants homozygous for pACA9-HyPer were selected in the subsequent F3 generation.

## Stable GFP-RbohH protein fusion expression in Arabidopsis pollen tubes

To generate the pACA9-GFP-RbohH constructs, full-length RbohH was amplified from WT cDNA flowers with the primer pairs RbohH-*Asc*I and RbohH-*Not*I using the Phusion® DNA polymerase (Finnzymes). The 2.7 Kb-long fragment was ligated into pJet1.2/blunt (Thermo Scientific), sequenced and then cloned into the *Asc*I/*Not*I cut binary ps1183 vector [ACA9-promoter-intron-GFP-CNGC16-STOP] replacing CNGC16 cDNA (Tunc-Ozdemir et al., 2013). Both WT and *rbohH-3 rbohJ-3* partially sterile plants were transformed with the pACA9-GFP-RbohH construct. Forty four out of 50 *rbohH-3 rbohJ-3* independent Hygromicin-resistant T1 lines showed rescue of fertility and exhibited similar expression pattern. Similarly, 49 independent T1 lines were obtained in the WT background. Three independent strong GFP-RbohH expressers T1 lines in WT and *rbohH-3 rbohJ-3* backgrounds were used for *in vitro* pollen germination and growth assays with similar outputs.

**Supporting References**

Boavida LC, McCormick S (2007) Temperature as a determinant factor for increased and reproducible *in vitro* pollen germination in *Arabidopsis thaliana*. Plant J 52: 570–82.

Boisson-Dernier A, Roy S, Kritsas K, Grobei MA, Jaciubek M, Schroeder JI, Grossniklaus U (2009) Disruption of the pollen-expressed *FERONIA* homologs *ANXUR1* and *ANXUR2* triggers pollen tube discharge. Development 136: 3279–3288.

Iwano M, Entani T, Shiba H, Kakita M, Nagai T, Mizuno H, Miyawaki A, Shoji T, Kubo K, Isogai A, et al. (2009) Fine-tuning of the cytoplasmic Ca^2+^ concentration is essential for pollen tube growth. Plant Phys 150: 1322–34.

Huck N, Moore JM, Federer M, Grossniklaus U (2003) The *Arabidopsis* mutant *feronia* disrupts the female gametophytic control of pollen tube reception. Development 130: 2149–59.

Hruz T, Laule O, Szabo G, Wessendorp F, Bleuler S, Oertle L, Widmayer P, Gruissem W, Zimmermann P (2008) Genevestigator v3: a reference expression database for the meta-analysis of transcriptomes. Adv Bioinformatics 2008: 420747.

Schiøtt M, Romanowsky SM, Baekgaard L, Jakobsen MK, Palmgren MG, Harper JF (2004) A plant plasma membrane Ca^2+^ pump is required for normal pollen tube growth and fertilization. Proc Natl Acad Sci USA 101: 9502–7.

Tunc-Ozdemir M, Rato C, Brown E, Rogers S, Mooneyham A, et al. (2013) Cyclic nucleotide gated channels 7 and 8 are essential for male reproductive fertility. PloS one 8: e55277. doi:10.1371/journal.pone.0055277.
